# Application of molecular dynamics simulation in self-assembled cancer nanomedicine

**DOI:** 10.1186/s40824-023-00386-7

**Published:** 2023-05-04

**Authors:** Xueli Xu, Ao Liu, Shuangqing Liu, Yanling Ma, Xinyu Zhang, Meng Zhang, Jinhua Zhao, Shuo Sun, Xiao Sun

**Affiliations:** 1 School of Science, Shandong Jianzhu University, Jinan, 250101 China; 2 School of Chemistry and Pharmaceutical Engineering, Medical Science and Technology Innovation Center, Shandong First Medical University & Shandong Academy of Medical Sciences, Jinan, 250000 China; 3 Department of Biostatistics, Harvard T.H. Chan School of Public Health, Boston, 02115 USA

**Keywords:** MD simulation, Nanomedicine, Self-assembly, Cancer Nanotheranostics

## Abstract

Self-assembled nanomedicine holds great potential in cancer theragnostic. The structures and dynamics of nanomedicine can be affected by a variety of non-covalent interactions, so it is essential to ensure the self-assembly process at atomic level. Molecular dynamics (MD) simulation is a key technology to link microcosm and macroscale. Along with the rapid development of computational power and simulation methods, scientists could simulate the specific process of intermolecular interactions. Thus, some experimental observations could be explained at microscopic level and the nanomedicine synthesis process would have traces to follow. This review not only outlines the concept, basic principle, and the parameter setting of MD simulation, but also highlights the recent progress in MD simulation for self-assembled cancer nanomedicine. In addition, the physicochemical parameters of self-assembly structure and interaction between various assembled molecules under MD simulation are also discussed. Therefore, this review will help advanced and novice researchers to quickly zoom in on fundamental information and gather some thought-provoking ideas to advance this subfield of self-assembled cancer nanomedicine.

## Introduction

### Challenges of cancer therapy

Over recent years, cancer has been one of the leading causes of death due to its high incidence and mortality [1]. Despite the increasing availability of antitumor regimens, patients’ quality of life and survival rate are still limited by the unfavorable side effects of existing treatments [2, 3]. Above all, current therapeutic approaches cannot meet the basic needs of patients. Therefore, the development of new drugs and the optimization of existing ones are the primary objectives. And the innovation of novel therapeutic with high efficiency and biosafety are the main focuses of cancer treatment, which has led to a shift in research towards nanomedicine [4]. The structures and dynamics of nanomedicine can be affected by a variety of non-covalent interactions, so it is essential to ensure the self-assembly process at atomic level. Molecular dynamics (MD) simulation is a key technology to link microcosm and macroscale. Along with the rapid development of computational power and simulation methods, scientists could simulate the specific process of intermolecular interactions. Thus, some experimental observations could be explained at microscopic level and the nanomedicine synthesis process would have traces to follow [5, 6]. Many MD simulation tools have been developed. For example, Lammps, DL_POLY and Material Studio are commonly used in the simulation of materials related dynamics, while Gromacs, Amber, NAMD and CHARMM are widely applied in the simulation of biological systems related dynamics.

Currently, with the development of science and technology, computer technology has been involved in all fields of life [7]. In the medical field, computers not only supply storage databases, but also play a huge role in structure-based drug design. This application of computer technology has greatly promoted the development of medicine [8]. Through traditional methods, it is a tricky problem to accurately confirm the successful assembly of two drugs. Also, the analyzation of biocompatibility and therapeutic effect at the molecular level is almost unachievable. With computer technology, these problems would be solved smoothly. For example, MD simulation has shown promising application prospects in the research fields of cancer, inflammation and other diseases, and has become an important support for improving clinical diagnosis and treatment [9]. Generally speaking, the development of a rare drug has only a 6.1% success rate, as for anticancer drugs, the rate could even be as low as 3.3%. Therefore, there is a growing commercial demand for computer simulation-assisted drug design to improve the efficiency of drug innovation [10].

### Self-assembled nanomedicine

Nanomedicine is the application of nanotechnology in medicine [11]. The formation and development of nanomedicine has brought great changes to the field of biology and medicine areas [12]. Nano-drug is at the forefront of nanotechnology in the twenty-first century. Compared to conventional nanotechnology, nanomedicine has a wider range of sizes and thus serves as a bridge between the macroscopic and the microscopic. Traditional nanomedicine is mainly built on nanomedicine carriers, which are diverse and complex, resulting in low drug loading capacity, complex preparation, lack of repeatability and controllability, and difficulty in mass production and clinical transformation. The self-assembled nanomedicines introduced in this paper take one or more drugs as the building unit, and realize self-assembly with other drugs or components through specific interactions by utilizing the structural properties of the drugs themselves. It is a carrier-free strategy. Self-assembled nanomedicine can greatly reduce the usage of the carrier, improve the encapsulation rate and drug loading capacity. Overall, there are two common nanomaterial-based drug delivery methods: one is carrier-based method, which consists of drug molecules and carriers such as polymers, lipids and carrier proteins. However, the carrier-based delivery would be the requirement of use of "excess" amount of synthetic material, which may lead to serious toxicity [13]. Another approach is the carrier-free strategy. The self-assembled nanomaterials introduced in this paper take one or more drugs as the building unit and realize self-assembly with other drugs or components through specific interactions by utilizing the structural properties of the drugs themselves. These self-assembled nanomaterials may have excellent bioavailability and drug delivery efficiency. Moreover, this material is simple to prepare with high controllability and good stability, and is expected to promote the large-scale production and clinical transformation of nanomedicine.

According to current researches, nano-drugs are mainly designed for three purposes: to improve drug solubility and internal absorption, to enhance drug targeting ability, and to act as special carriers for some biological macromolecules [14]. For example, non-specific drug could be loaded in nanospheres and delivered precisely to target cells [15]. For some drugs that lack the ability to target, by coupling small molecules with the ability to target that self-assembled into a new nano drug to endow the ability to target. There are also some targeted drugs that are hydrophobic or have high toxicity, and then self-assembly with other small molecules can not only prolong blood circulation time, but also improve biocompatibility. For example, Du et al. [16] co-assembled the epidermal growth factor targeting peptide with Gemcitabine (Gem). The assembled nanodrugs not only have good biocompatibility and high drug encapsulation rate, but also realize the ability to actively target tumors through the recognition of the targeting peptide, avoiding systemic toxic effects. Lan et al. [17] formed uniform stable nanomaterials (GA-Ce6-FA) by self-assembly of luteic acid, Ce6 and folic acid (FA). The assembled nanomaterials could achieve tumor site accumulation through EPR effect, and improve drug concentration at tumor site through tumor targeting of FA. Effective treatment of tumors has been achieved. Lei et al. [18] formed copolymers through electrostatic forces between polyglutamic acid (PGA), PTX and Gem, and then used biodegradable cationic dendrimers (PLDS) and FA as self-assembly initiators to form uniform and stable nanomaterials. The nanomaterials show good structural stability and certain inhibition ability of tumor cell proliferation. Meanwhile, the participation of FA enables the nanomaterials to have the targeting ability and improve the accumulation ability at the tumor site.

The in-depth study of self-assembly has held the attention of increased researchers. For the construct of nanomedicine, molecular self-assembly is a rising strategy and has been widely used [19]. The mechanism behind self-assembly is the formation of non-covalent bonds include hydrophobic interaction, π-π stacking, electrostatic interaction, and hydrogen bonding between molecules. In other words, self-assembly is a spontaneous aggregation process among random moving molecules in liquid phase [20–22]. Moreover, the emergence of supramolecular nanomaterials further promotes the application of self-assembly in tumor treatment [23, 24]. Self-assembled nanomedicine can integrate the advantages of multiple functions, which can not only improve the efficiency of tumor treatment, but also can improve their imaging ability. Currently, the commonly used contrast agents are often small molecules. However, due to the lack of tumor tissue specificity, stability and tissue penetration, these small molecules are often quickly eliminated by the blood, which limits their application in clinical tumor imaging. Self-assembly of imaging molecules and other small molecules can improve their stability and penetration ability, which can achieve enhanced imaging ability [25, 26]. For example, An et al. coupled amino acid polypeptides with cyanine-like dye molecules, and can form fibrous morphology by apoptosis suppressant protein mediated self-assembly. It found that nanoparticles showed enhanced accumulation in tumor areas, effectively improving the permeability and imaging ability of fluorescent molecules in tumor tissues [27]. Dong et al. realized the self-assembly of Ce6, PTX and IR783 through hydrophobic forces to construct a theranostic nanodrug (Ce6-PTX@IR783). This nanodrug with good biodegradability had ultrasound-activated sonodynamic therapy, PTT, and photoacoustic imaging performance [28].

In general, self-assembled nanomedicines play an important role in anticancer treatment because they are programmable, biocompatible and multifunctional [29]. However, there are also obstacles to the self-assembled nanomedicines drugs into the clinic [30]. Firstly, carriers applied in self-assembly are usually so large that the drug loading capacity would be restricted. Secondly, the process of assembly is complex at the microscopic level and difficult to control [31]. With the development of science and technology, the precise mechanisms behind the self-assembly of molecules have attracted widespread attention. To explore the mechanism of molecular self-assembly, simulation tools are required. Among all the tools available, MD simulation stands out for its ability to connect the macro and the micro. What’s more, its runtime could be as short as microsecond level. Therefore, MD simulation, which visualizes the assemble process of molecules, provides an effective simulation platform to study and assess self-assemble at nanoscale.

### MD simulation

MD simulation, also known as computer experiment, is to obtain the relative motion trajectory of an atom or molecule based on the principle of Newton's equations of motion, and then obtain the required kinetic property parameters according to the trajectory [5, 6]. MD simulation focuses on many-body systems including nuclei and electrons, the motions of nucleus are simulated by computer, thereby obtain the conformations and properties of the system. Then the micro and macro systems are connected by computer assistance. This process spans many disciplines including physics, chemistry, and biology, many other fields will be involved and play a specific role [32]. MD simulation has generally been known to the public as a newly emerging technology. However, the history of MD simulation could date back to the 1940s. The first gas MD simulation was carried out in 1957 and the first protein MD simulation was published in 1968 [33]. Recently, with the continuous improvement of computing power and simulation algorithms, MD simulation has become an indispensable research method in many fields [34].

#### Basic principles of MD

The basic theory of MD is evolved from Newton's second law [35]. In a motion system full of particles, the total energy is the sum of the kinetic and potential energy of each particle. Assuming the atoms in the system move according to a definite description, Newton’s second law is used to connect the motion of the atoms with the resulting trajectory. In brief, MD determines the potential energy of an atom from the coordinates of its individual atoms and then uses the potential energy to predict the position of the atom at next moment, making its position heritable through statistical methods [36]. Therefore, when simulating with MD, the most important thing is to determine the potential interaction between atoms. The most used potential functions are the Lennard–Jones potential and Buckingham potential [37].

In combination with the motion trajectory, MD simulation is based on the motion law of each particle in the simulation system described by Newton's law of motion [38]. The motion equation of the particle is:

$$F_{\mathrm i}=\frac{\mathit\partial U}{\partial r_{\mathrm i}}=\frac{m_{\mathrm i}\partial v_{\mathrm i}}{\partial t}=\frac{m_{\mathrm i}\partial^2r_{\mathrm i}}{\partial t^2}$$

where ${\mathrm{m}}_{\mathrm{i}}$, ${\mathrm{r}}_{\mathrm{i}}$ ${\mathrm{v}}_{\mathrm{i}}$, express the mass, position, and velocity of the particle, respectively. The functions ${\mathrm{F}}_{\mathrm{i}}$ and U are the resultant force on the particle and the total potential energy of the system, respectively. Through this formula, the force on each particle is calculated, combined with the current speed and position of the particle, the position and velocity of the particle at the next moment $\mathrm{\Delta t}$ is estimated, and the trajectory of particles in the whole system can be obtained [34]. The trajectory is dynamic, which Includes the information of all the particles’ changing over time, location, speed, and force. The choice of $\mathrm{\Delta t}$ mainly relies on the type of simulation system and the level of refinement described.

#### Force field

Force field selection is very important in MD simulation process [39, 40]. Force field is a set of function sets of the internal coordinates of the particles in the system. As a variable quantity, it can be used for modelling system [41]. The variability of the force field is mainly determined by the difference between the potential function and the structural parameters of the force field. With the continuous development of computer technology, many MD simulation force fields have been established and the simulation algorithms are constantly being improved. Therefore, MD simulation has also been promoted to receive more attention [34]. Although most of the force fields contain the same potential energy function, there are still some force fields with different potential energy functions for different treatments. Take the interaction between non-bonds as an example, which is obtained by full system integration at each step size. Because of the large amount of computation, truncation is generally adopted. But nonbonded interactions belong to short-range interactions that decay as the inter-particle distances increase. Therefore, more smooth transitions are adopted in practical practice [42].

At present, the commonly used force fields in biomedicine mainly fall into the following three categories: The first category is All-atom force fields [43]: Such as assisted model build-ingand energy refinement (AMBER), optimized potentials for liquid simulations (OPLS) and chemistry at harvard macromolecular mechanics (CHARMM) etc., this kind of force field is characterized by high accuracy. Every atom of the molecule corresponds to one of the force points in the simulation system, which can better describe the biomolecular system. However, the calculation amount required for simulation is huge and the simulation efficiency is low. Specifically, AMBER force field is suitable for processing small biochemical molecules such as proteins, nucleic acids and polysaccharides, and all the calculated results of force field parameters are derived from experimental values [44]. CHARMM force field is applied to small organic molecules, solutions, polymers, biochemical molecules, and other molecular systems. The calculation accuracy of hydrogen bond energy is high, and the bond length and bond Angle of hydrogen bond can be controlled. The calculation results of force field parameters are not only from experimental values, but also refer to the quantum calculation results of large [45]. OPLS force field is applicable to liquid system, mainly applied to polypeptide, protein, nucleic acid, organic solvent and other liquid system, generally applicable to the water model of Tip3P or TIP4P, focusing on the description of condensed phase properties [46].

The second category is United-atom force fields [47]: The GROMOS force field is the most representative one. This kind of force field is characterized by the fact that carbon atoms and hydrogen atoms directly bonded to carbon atoms are regarded as whole joint atoms, and the interactions of other atoms on hydrogen atoms are also superimposed on the joint atom, greatly reducing the complexity of force field and potential parameters. GROMOS 'field parameters are mainly obtained by fitting experimental data with thermodynamic characteristics of pure fluid or mixed fluid systems in condensed matter, which is mainly applied to biomolecular systems [48].

The third category is Coarse-grained force fields [49]. It is much simpler and more abstract than the first two types of simulation systems [50]. The coarse-grained molecular force field treats multiple heavy atoms and their connected hydrogen atoms as a whole unit, and increases the time and space scale of simulation by simplifying the number of particles in the system [51–53]. For example, the benzene ring and its hydrogen bond can be viewed as a whole, thus reducing the complexity of the system. The common force field is MARTINI force field, which mainly expresses biological macromolecular system and is widely used in the research fields of protein folding and surfactant aggregation.

#### Ensemble and boundary condition

Ensemble and boundary conditions are the most basic parameters in MD simulation. The choice of these two parameters determines the fluctuation of thermodynamic energy and computation of the system [54]. MD is performed under certain conditions, because of equivalence, the thermodynamic quantities of each system are the same under different fluctuations. But different systems could cause diverse thermodynamic quantities. According to macroscopic conditions, ensembles are divided into microcanonical, canonical (NVT), isothermal and isobaric (NPT) and isothermal enthalpy ensembles [55, 56].

Boundary conditions in MD simulation are key factors for keeping the system constant and solving the "size effect" in simulation process [57]. When there are too many particles in the simulation system, the simulation time will be too long to analyze, so the number of analysis objects should be smaller than the actual particles. On this basis, MD simulations can set boundary conditions for various systems to reduce the effect of finite size on MD simulation experiments [58]. There are two main types of boundary conditions in MD simulation: isolated boundary conditions and periodic boundary conditions [59].

#### Simulation software

Having developed so far, various types of simulation software is emerging, now widely used software is Groningen Machine for Chemical Simulations (GROMACS), AMBER, CHARMM and Nanoscale Molecular Dynamics (NAMD). The primarily chosen simulation software in the field of nanomedicines is GROMACS. GROMACS incorporates almost all current popular algorithms for MD simulations so it is dominating in performing a large number of calculations (such as biological macromolecules) [60]. At the same time, the software also embeds amount of data analysis tools which is more convenient to use. The comparison of the advantages and disadvantages of various simulation software is in Table 1.

**Table 1:** Comparison of various simulation software

Name	Research Direction	Advantage	Disadvantage	Ref
NAMD	Simulated organism, chemical soft materials	Compatible with multiple file formats, best parallel processing and high efficiency	Restrictions on metal atoms	[61]
AMBER	Biological system, few chemical system	Convenient modeling of new molecules and models	Low computational efficiency and slow speed	[62]
CHARMM	Biological system, few chemical system	Fast updating potential energy model	Low computational efficiency and slow speed	[63]
GROMACS	Bioprotein system	Support all simulation algorithms	No suitable to research abiotic protein field	[64]
LAMMPS	Material, Metal field	High parallel processing efficiency, complete ensemble and potential functions	Data processing function is weak	[65]

In summary, besides the ability to obtain general characteristics and interesting behaviors of the simulated system, MD simulation could observe and display experimental procedure just like real experiments. Especially, many microscopic and atomic scale details which cannot be obtained in traditional experiments, can be easily observed in MD simulation. This advantage makes MD very attractive in several fields including biomedicine. With the continuous improvement of computer performance, AB Initio calculation of MD without empirical potential function will not only achieve more accurate calculations but also have a wider range of applications. Therefore, AB Initio MD will become the main development direction of MD simulation in the future. In this review, we will launch a new field for readers, comprehensively introduce the concept, principle, and current development trend of MD simulation. Furthermore, the latest application of MD simulation will also be explored in tumor therapy in self-assembled nano-drugs.

## Application of MD simulation in self-assembled anticancer nanomedicine

**Fig. 1:**
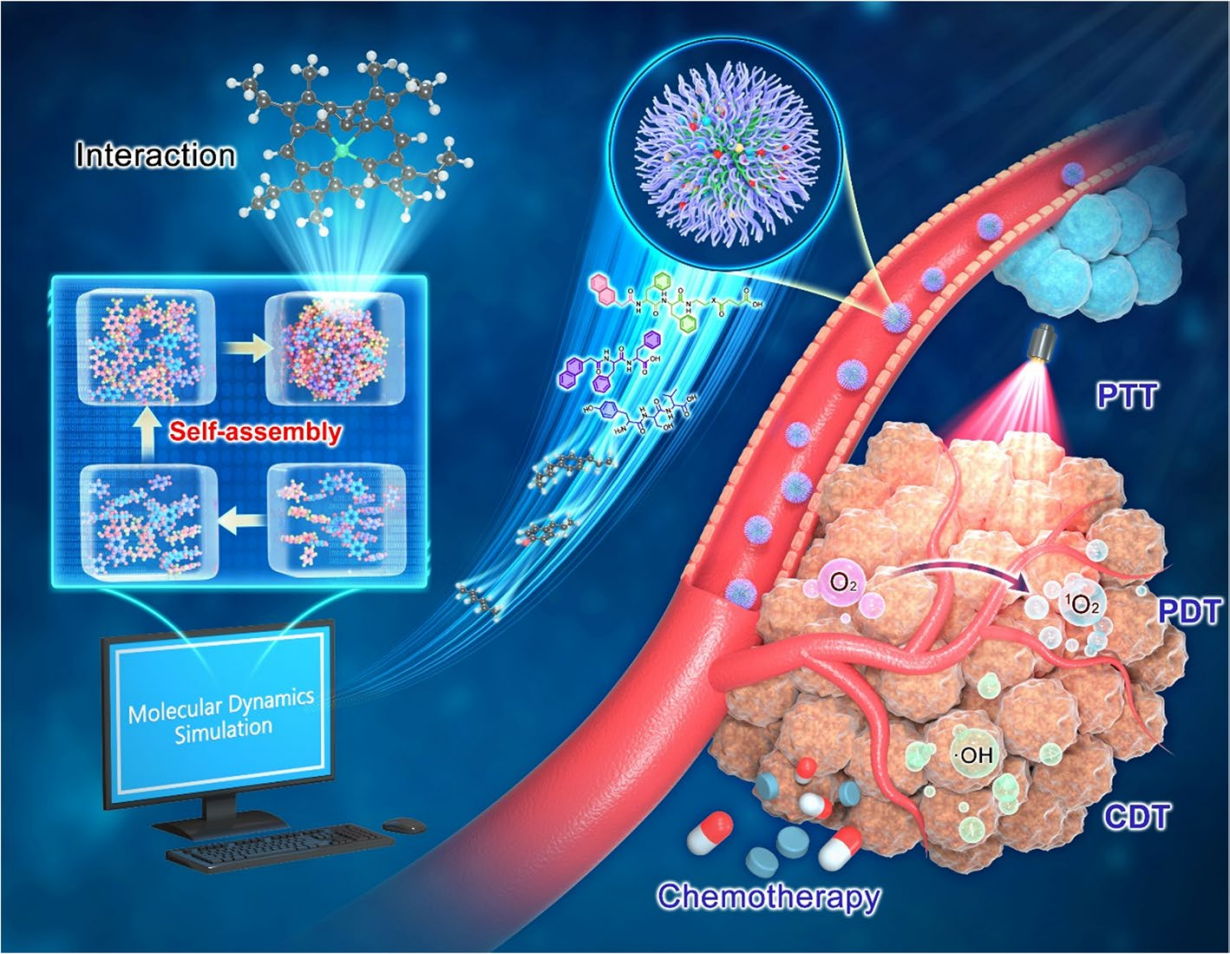
MD simulation for self-assembled cancer nanotheranostic application

Over recent years, the combination of MD simulation and experiment has attracted increasing attention in many areas including medicine, biology, chemistry, materials, machinery, and new energy. Scientists could dig out accurate data and information from precise analysis, and provide clues about the changing trends of molecules. In medicine, MD simulation for self-assembled cancer nanotheranostic application is shown in Fig. 1. Examples of MD simulation in nanomedicine researches are listed in Table 2.

**Table 2:** Application of MD simulation in nanomedicine

Material composition	Composition shape	Simulation software	Force field	Treatment	Tumor types	Ref
Doxorubicin, Chitosan	chain	GROMACS	AMBER (GAFF)	Chemotherapy	---	[66]
Estrogen-related receptor α	Spiral	AMBER	ff14SB	Chemotherapy	---	[67]
Triazine-morpholino derivatives	Chain	AMBER	AMBER7FF9	Chemotherapy	Breast cancer	[68]
Genistein, AR endogenous agonist DHT	Spiral chain	GROMACS	OPLS-AA	Chemotherapy	Prostate cancer	[69]
Fullerenes, gamma-cyclodextrin; inclusion complexes	Ring	Material Studio	CVFF(Consistent Valence-Force Field)	PDT	---	[70]
Levonorgestrel	Spiral chain	GROMACS	AMBER03	Chemotherapy	Breast cancer	[71]
Mammalian target of rapamycin	Chain	GROMACS	AMBER7FF99	Chemotherapy	Lung cancer	[72]
DOX, PAX	Nanotube	GROMACS	OPLS-AA	Chemotherapy	Lung cancer, breast cancer	[73]
Anodic aluminum oxide, tantalum nitride-coated Si	Nanodot array	GROMACS	---	Chemotherapy	---	[74]
Catalase-like RuO_2_@BSA@IR-808-Br2(RBIR) nanozyme	Nanotube	GROMACS	---	PTT PDT	Breast cancer	[75]
HCPT/Ce6 NRs	Nanorod	GROMACS	GROMOS53A6	PDT	Breast cancer	[76]
Epidermal growth factor receptor	Chain	AMBER	GAFF(Generation Amber-Force Field)	Chemotherapy	Lung cancer	[77]
styrylquinolines	Chain	NAMD	CHARMM27	Chemotherapy	---	[78]
Hsp90 C-terminal domain inhibitors	Chain	NAMD	CHARMM22	Chemotherapy	---	[79]
SN38, trolox, succinic acid linker	Nanoparticles	GROMACS	OPLS-AA	Chemotherapy	Colorectal cancer	[80]
TH peptides	Cyclic peptide	GROMACS	AMBER, OPLS-AA	Chemotherapy	Brain tumor	[81]
Pure drug nanoa-ssemblies	Nanoparticles	GROMACS	---	PTT, PDT	---	[11]
Daunorubicin, Etoposide	Nanotube	Gromacs5	CHARMM27	Chemotherapy	---	[82]
DOX, Aluminum Hydroxide	Nanosheet	LAMMPS	CHARMM27	Chemotherapy	Melanoma	[83]
Cu (II)-Schiff base complexes	Nanoparticles	AutoDock4	---	Chemotherapy	Human erythroleukemia cancer	[84]
Aptamer -receptor	Nanoparticles	GROMACS	CHARMM	Chemotherapy	Breast cancer	[9]
Adriamycin, chitosan, graphene	Nanosheet	GROMACS 5.0.7	---	Chemotherapy	---	[85]
P2Y1R receptor - indoline-4 carbonitrile HIC	Spiral chain	GROOMACS 4.6	GROMOS	Chemotherapy	Prostate cancer	[86]
HDAC inhibitors	Nanoparticles	NAMD	CHARMM	Chemotherapy	Prostate cancer	[87]
DOX, carbon nanotubes	Nanotube	AMBER	CHARMM36	Chemotherapy	---	[88]
DOX, black phosphorus	Nanosheet	GROMACS	CHARMM	Chemotherapy	---	[89]
Selective estrogen receptor downregulators（SERDs）	Reticular	AMBERTOOLS	---	Chemotherapy	Breast cancer	[90]
FPTT	---	GROMACS	OPLS	Chemotherapy	Breast cancer	[91]
RET kinase	Crystal, structure	AMBER18	AMBER	Chemotherapy	Thyroid cancer	[92]
MTORC1, arginine analogues	---	AMBER16	GAFF/ff144SB	Chemotherapy	Lung cancer	[93]
Au NP, Panobinostat	Nanoparticles	AMBER19	---	Chemotherapy	---	[94]
SERDs	---	AMBERTPPLS14	AMBER	Chemotherapy	Breast cancer	[95]

### MD simulation with PTT

PTT converts NIR light energy into heat energy through a photothermal agent to raise tumor sites to more than 50 °C and rapidly destroy tumors [96, 97]. Since near-infrared light is a relatively safe laser, PTT has become a safe, effective anti-tumor therapy with less adverse effect. In recent years, PTT has received extensive attention in tumor ablation research and clinical fields [98]. However, due to the limited transmission ability of the laser and the uneven heat distribution during the irradiation process, PTT alone cannot effectively kill the tumor. To achieve the desired therapeutic effect, researchers often synergize PTT by combining with other treatments.

#### Self-assembled I_2_ nanomedicine

**Fig. 2:**
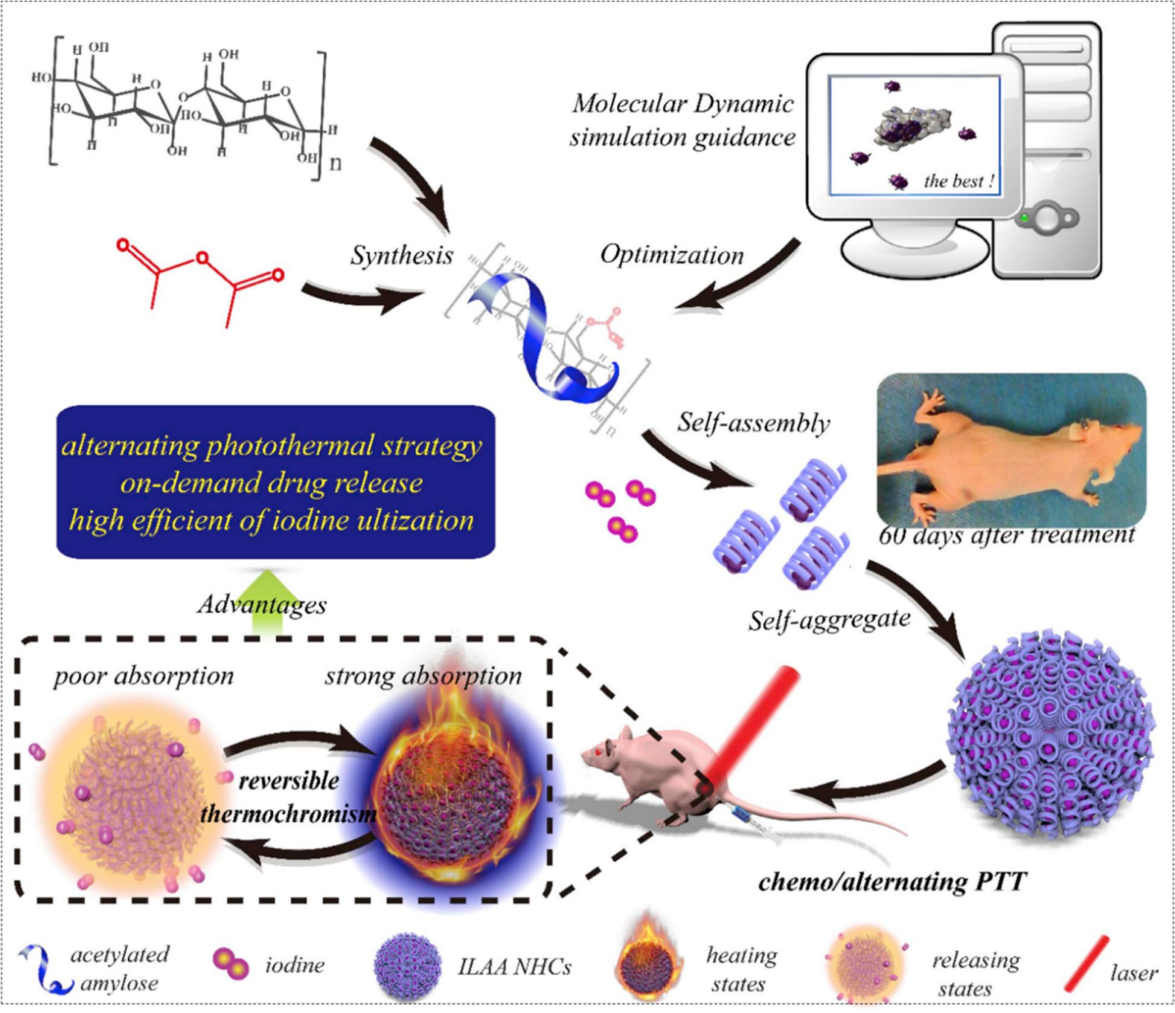
Synthesis and Therapeutic Protocol for ILAA NHC. Reproduced with permission from Ref. [99]

To ameliorate the defects of chemotherapy and PTT. Tang et al. [99]. designed an iodine (I_2_)-supported acetylated amylose nanohelix clusters (ILAA NHCs), a novel alternating photothermal system that induced temperature self-regulation by heat regulated color change. Guided by MD simulations, I_2_ was loaded into the helical cavity of acetylated amylose (AA) via hydrophobic interactions, and then self-assembled into nanoclusters. The synthesis and therapeutic protocol for ILAA NHC were shown in Fig. 2. The specific simulation was as follows, the initial structural simulation of the ILAA helix was established first. With the GROMACS 53A6 force field and the parameters were stabilized, the temperature was changed, and the structural evolution of the simulated 100 ns was observed. It was found that ILAA NHC performed multifunctional photothermal conversion through its unique reversible thermochromism with ultra-high photothermal depth. At the same time, I_2_ effectively played the dual role of chemotherapy and PTT. The results showed that ILAA NHCs had a good tumor treatment effect, and this synergistic chemotherapy/PTT approach was more effective in killing tumors than single chemotherapy or PTT. At the same time, this synergistic chemotherapy/PTT scheme with an alternating photothermal strategy had great potential in tumor treatment.

#### Self-assembled indocyaninegreen (ICG) nanomedicine

**Fig. 3:**
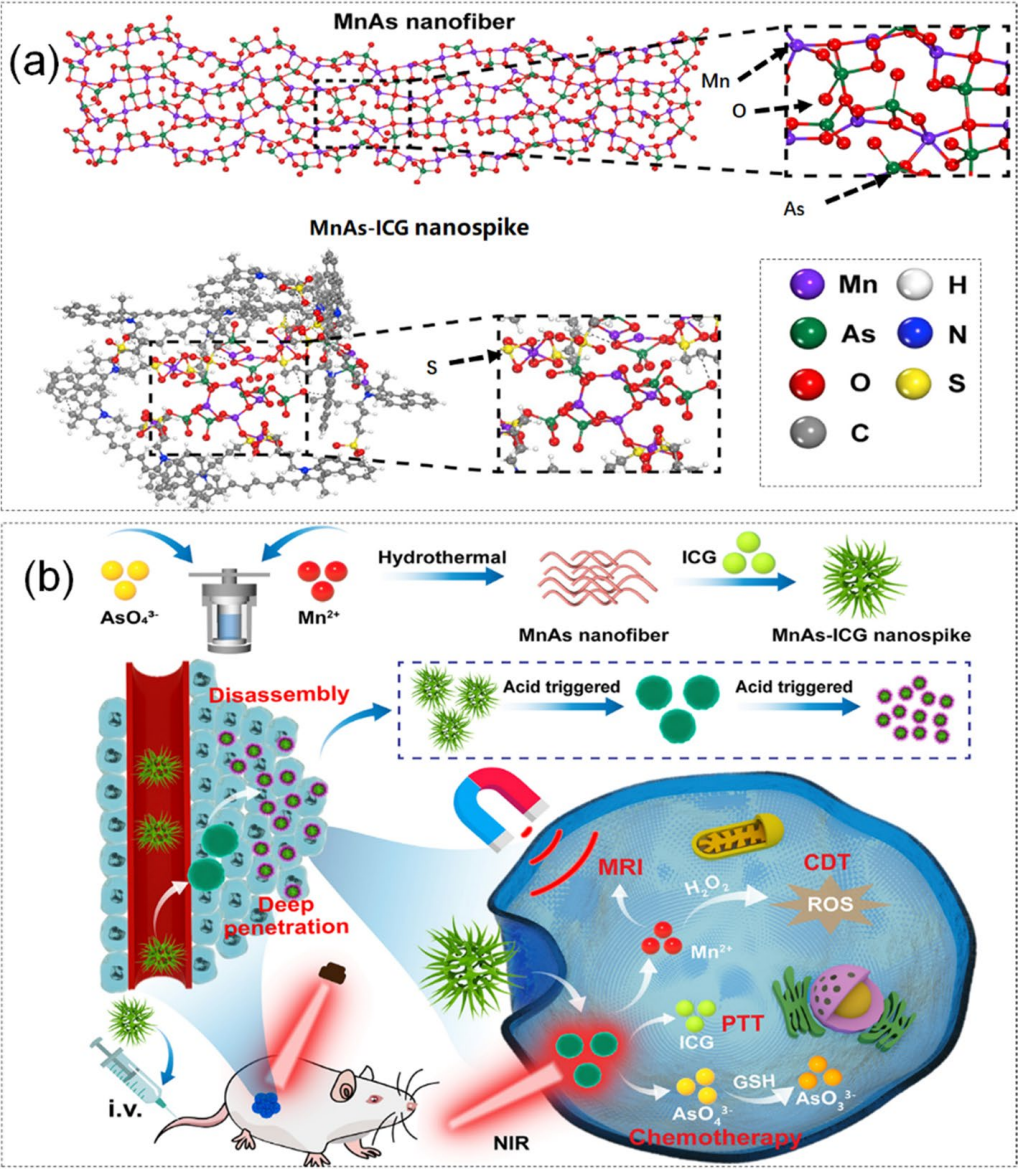
**a** MD simulation of MnAs-ICG nanospike. **b** Preparation process and tumor treatment scheme of MnAs-ICG nanomedicines. Reproduced with permission from Ref. [100]

In recent years, synergistic treatment of PTT with chemotherapy and Chemodynamic Therapy (CDT) has been shown to be feasible. Based on this, Chen et al. [100]. self-assembled indocyanine green, manganese ions, and arsenate into a novel nanomedicine, MnAs-ICG via intermolecular interactions. Thus prepared a fluorescence/magnetic resonance bimodal imaging guidance diagnostic nanoplatform to achieve the combined treatment of PTT/chemo/CDT. At the same time, researchers explored the interaction mechanism between the coordination of MnAs from nanofibers to nanospike after the addition of ICG by MD simulation. MNAS-ICG nano-texture molecular structure and its tumor treatment plan were shown in Fig. 3 (a, b). The specific simulation was as follows. Firstly, the conjugate gradient algorithm was used to obtain a stable structure, and the energy was minimized. Then, researchers maintained the equilibrium state of temperature and pressure under the NPT ensemble. Simulated 3000 ps to obtain the target characteristics and interaction strength.

The as prepared MnAs-ICG nanoparticles had uniform morphology, good loading capacity, high PTT conversion efficiency and excellent pH/PTT dual-responsive release. Compared with a single component, MnAs-ICG could release drug components according to its own needs with good biocompatibility, increase the accumulation of drugs in tumor sites, and effectively achieve the combined treatment of PTT/chemo/CDT. Altogether, this new nanoparticle held great promise for the rational design of multifunctional therapeutic nanoplatforms for breast cancer treatment.

#### Self-assembled TFM/GRN nanomedicine

**Fig. 4:**
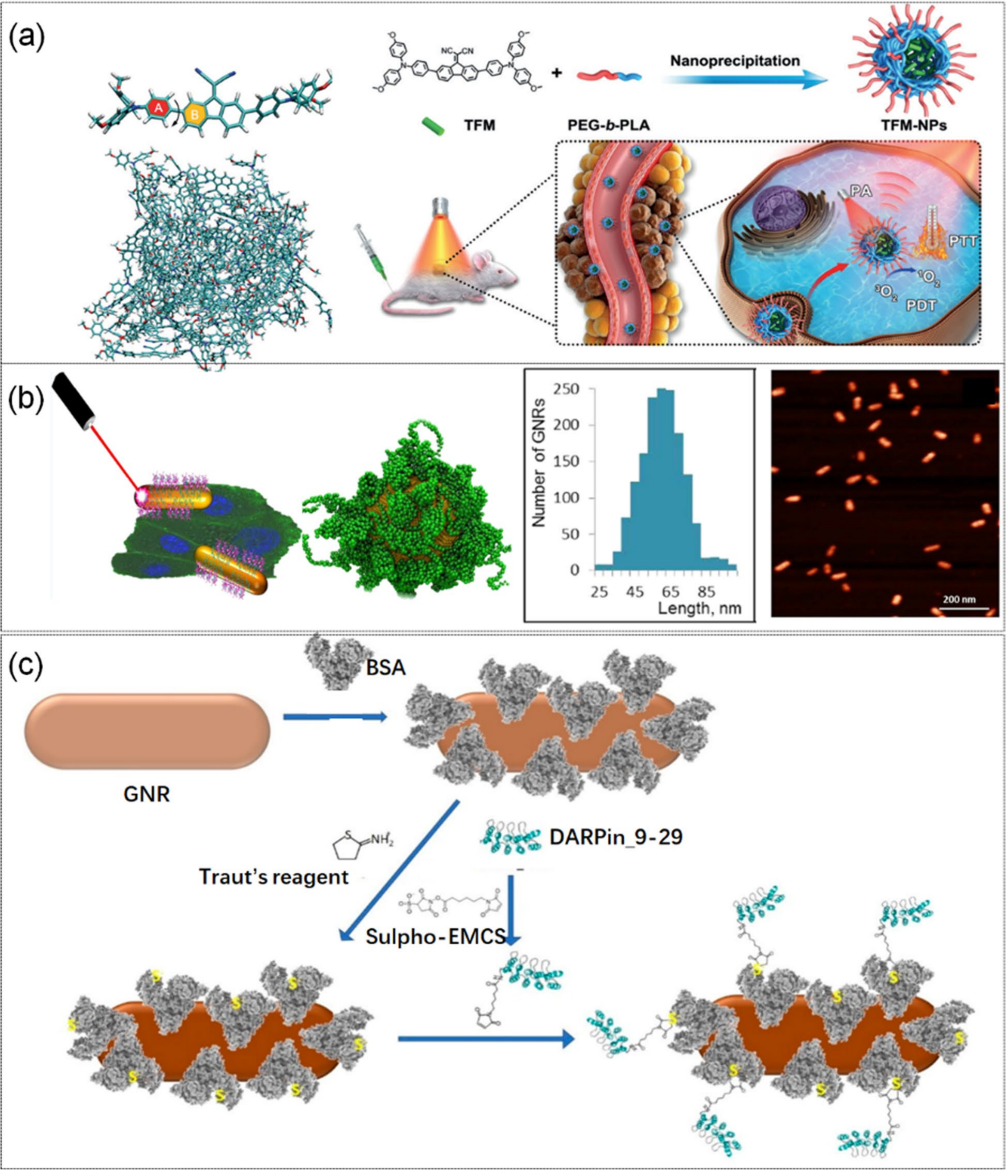
**a** Molecular structures of single TFM and TFM aggregates under MD simulation, and schematic diagram of the preparation and cancer treatment of TFM nanoparticles. Reproduced with permission from Ref [101]. **b** A schematic of tumor inhibition and structural images of the DARPin-coated nanorods under the front, and Atomic force microscopy and average size of DARP-GNRs. Reproduced with permission from Ref [102]. **c** DARPin-BSA-GNR synthesis scheme: The HER2-binding domain of the DARPin_9-29 molecule is shown in gray. Reproduced with permission from Ref [103]

Radiative decay, the energy dissipation pathway that accompanies luminescence, has fascinated scientists in recent years. Radiative decay is now applied as a key technology in many fields, including electronic sensing, bioimaging, and optical instrumentation. Researchers have also been working on suppressing nonradiative decay to improve luminous efficiency. As a chromophore, aggregation-induced emission (AIE)-active emitters (AIEgens), whose free rotation or vibration contributes to nonradiative decay, could generate substantial heat. The molecular structure and tumor treatment plan of a single TFM and TFM cluster was shown in Fig. 4 (a, b).

Based on this discovery, Wang et al. [101]. prepared a new compound (TFM) and explored intramolecular motions by MD simulation. In this simulation, the single TFM structure and the amorphous TFM aggregate structure were first constructed, and energy minimization was performed for the two systems, respectively. Then, the equilibrium state of temperature and pressure were kept under the NPT ensemble. The results showed that the compound was amorphous with a twisted spring structure and exhibited strong absorption ability under laser irradiation and AIE. Furthermore, TFM nanoparticles showed ultra-high photothermal conversion ability, good photoacoustic (PA) effect, and efficient reactive oxygen species (ROS) generation, making them ideal candidates for PA-induced PTT production.

In recent years, the development of gold micro-nanorods (GNRs) and polymers of nanorods and proteins with tunable optical properties have greatly pushed the application of nanomaterials in PTT. Conjugates of proteins and nanorods are nontoxic with strong NIR absorption. At the same time, ankyrin repeating protein (DARPin) and human epidermal growth factor receptor 2 (HER2) have high affinity interaction, which can specifically target HER2-positive cells, and DARPin as well as gold nanostructure conjugate can be specifically delivered to HER2-positive cells. Based on this principle, Galina et al. [102]. studied the assembly of the ankyrin repeat protein DARPin_9-29 with miniature gold nanorods to form a conjugate DARPin-GNR by CG-MD simulation. Firstly, the original structure of DARPin-HER2 polymer was downloaded from the website and an initial model of GNR was established. Then, maintaining the equilibrium state of temperature and pressure under the NPT ensemble. Three independent MD simulations were performed at equilibrium temperature by varying the start-up speed and coupling time. Structural images of the DARPin-coated nanorods under the front and side surfaces were shown in Fig. 4 (b). The simulation showed that 160 out of 250 DARPin- molecules adsorbed on the GNR surface, forming a single layer. Experiments showed that the surface of the nanorods was coated with a layer of protein, which had strong absorption and excellent biocompatibility. The DARPin receptor-binding domain that constitutes this layer was not involved in the self-assembly of the protein to the surface of the gold nanorods, and could induce the death of HER2-overexpressed human breast cancer cells under NIR irradiation. However, after many trials, the researchers found that the DarPin-coated nanorods tended to accumulate in blood vessels, making the coupling drug less effective against tumors in the body. In order to overcome the low colloidal stability of the conjugants, Galina et al. [103] coated the nanorods with bovine serum albumin (BSA) prior to DARPin coupling, which increased the colloidal stability and biocompatibility of the gold nanostructures. Meanwhile, Darpin-Bsa-GnRs can reduce the accumulation in the blood and increase the target towards HER 2-positive tumors. These properties made the conjugates suitable candidates for cancer PTT.

### MD simulation with PDT

PDT has developed rapidly in the past decade. PDT uses cell-specific chemical reactions to generate cytotoxic ROS to destroy biomolecular structures and induce tumor cell necrosis [104, 105]. PDT has good therapeutic effect on superficial tumors such as skin cancer and head and neck cancer, so it has gradually become the mainstream of tumor treatment to supplement the surgical treatment [106]. Due to the multi-level complexity and variability of advanced cancers, PDT is unable to meet the demands along, therefore, the combined treatment strategies based on chemotherapy and PDT are widely used in cancer treatment [107].

Photosensitizer is the basic and important factor in PDT, the research for photosensitizer has been paid an increasing attention [108]. Chlorin e6 (Ce6), a photosensitizer extracted from chlorophyll, is a single structure with low cytotoxicity and the ability to generate a large amount of ROS [109]. Although Ce6 has properties close to the ideal photosensitizer, the water insolubility has raised a bar for researchers to make Ce6 loading nanomedicine [110].

#### Self-assembled Ce6 nanomedicine

**Fig. 5:**
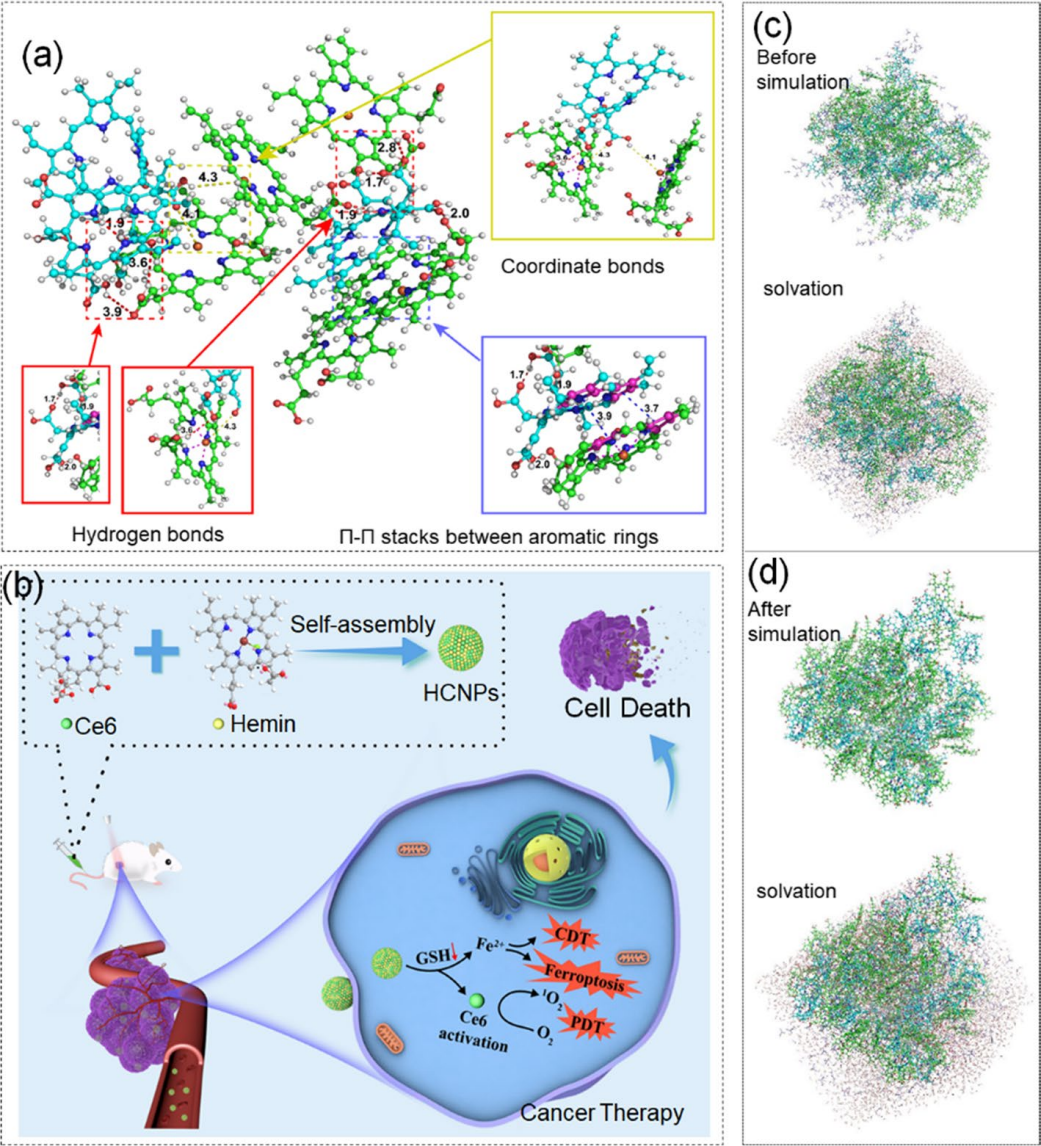
**a** Structural simulation of HCNPs under MD simulation. **b** Schematic diagram of the preparation process and tumor treatment of HCNP nanoparticles. The initial conformation and solvent formation of Hemin, Ce6 and DMSO (**c**) before and (**d**) after simulation. Reproduced with permission from Ref [112]

The combination of PDT, CDT and ferroptosis is also an importance approach to enhance anticancer effect [111]. Chen et al. [112]. used MD simulation to design a nanoparticle (HCNP) self-assembled from Ce6 and Hemin molecules. Under MD simulation, the HCNP molecular structure, preparation process and tumor treatment mechanism were shown in Fig. 5 (a, b). The specific simulation was as follows, the charges of Ce6 and dimethyl sulfoxide were generated by GAFF. The initial model was built by packmol. After energy minimization, maintained the equilibrium state of temperature and pressure under the NPT ensemble. Initial conformation and solvated water structure of Hemin, Ce6 and DMSO before and after simulation were shown in Fig. 5(c, d). In this study, HCNP could induce the decomposition of hydrogen peroxide (H_2_O_2_) inside tumor cells to generate ^1^O_2_ to achieve the effect of killing tumors [113, 114]. And the occurrence of the Fenton reaction could decompose H_2_O_2_ into highly oxidized hydroxyl radicals (•OH) and oxygen (O_2_) and destroy the cellular oxidation by consuming intracellular glutathione (GSH). Furthermore, the restored homeostasis, enhanced the ability of CDT, depleted intracellular GSH, and blocked GPX4 activity had not only further enhanced the ability of PDT/CDT, but also induced ferroptosis. The synergistic treatment of ferroptosis/PDT/CDT/ significantly achieved the strongest antitumor effect under NIR irradiation.

In PDT, hypoxia is a key feature of tumor microenvironment (TME) and to increase O_2_ production is a promising method to enhance PDT [115, 116]. The increase of the concentration of ROS and O_2_ in TME is the current problem for enhancing PDT. Fortunately, researchers found that H_2_O_2_ can undergo a Fenton-like reaction with iron ions to produce O_2_ [117, 118]. Therefore, researchers envision whether Fenton-like reaction can be combined with PDT to improve the therapeutic efficiency of PDT [119, 120]. Ferroptosis is an iron ions induced process in which GSH peroxide 4 (GPX4), could consume intracellular GSH and generate excess ROS to induce cell death [121, 122]. ROS generated by ferroptosis will be continuously converted into O_2_ by the Fenton-like reaction, further improving the efficacy of PDT [123, 124].

**Fig. 6:**
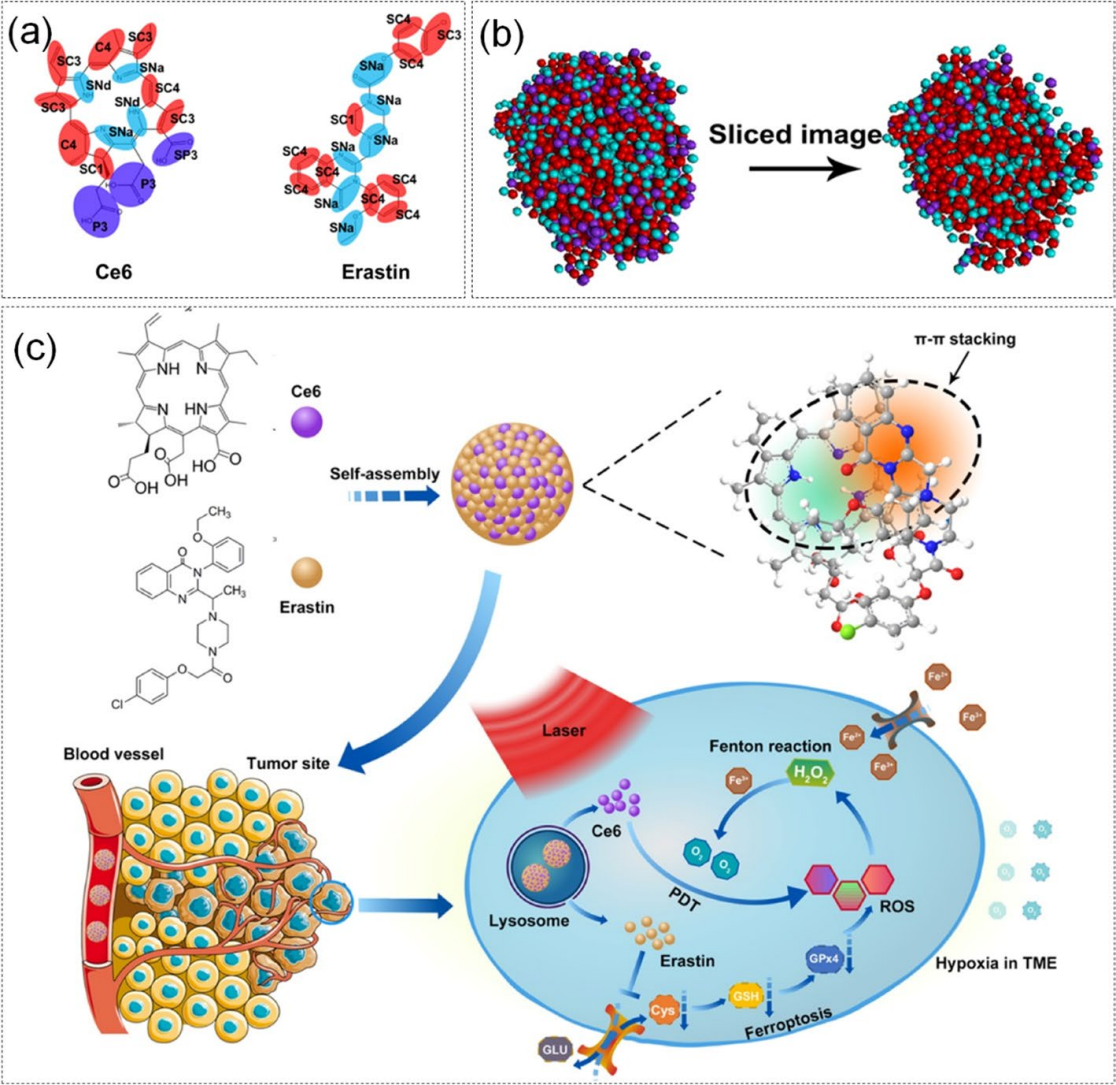
**a** CG model component diagram of Ce6 and erastin. **b** Overall molecular structure diagram (front and side) of Ce6-erastin nanoparticles. **c** Schematic diagram and mechanism study of Ce6-erastin nanoparticles. Reproduced with permission from Ref. [125]

Zhu et al. [125]. proposed a new anti-cancer treatment by combining ferroptosis and PDT. Self-assembly between photosensitizer Ce6 and ferroptosis stimulator erastin under MD simulation is the core of this study. The coarse granulation models and overall molecular structure diagrams of Ce6 and Erastin are shown in Fig. 6 (a, b). The specific simulation was as follows, the MARTINI force field was applied to perform CG-MD simulation on Ce6 and erastin. The initial model was built and optimized by L-BFGS algorithm, maintained the equilibrium state of temperature and pressure under the NPT ensemble, and finally, CG-MD of 300 ns was used to explore the properties of nanoparticles. Experiments showed that Ce6-erastin nanoparticles had good biocompatibility and could induce ferroptosis to continuously generate O_2_ through the Fenton reaction under light. Furthermore, this process could generate more ROS and lead to the oxidation of cellular components damage, thereby promoting the efficacy of PDT therapy. The treatment plan for Ce6-erastin nanoparticles was shown in Fig. 6 (c). The ferroptosis-promoting PDT approach significantly enhanced anticancer effects by alleviating hypoxia, and promoted ROS production to provide a novel idea to surmount hypoxia-related drug resistance of PDT.

The natural alkaloid camptothecin (CPT) and SN38 have anti-tumor effects as topoisomerase I inhibitors and are priority of chemotherapeutic drugs [126]. SN38 is one of the most active analogs, has poorer solubility than other analogs including Hydroxycamptothecin (HCPT), which greatly limit its clinical application [127]. Irinotecan hydrochloride (CPT11) is a soluble prodrug derived from CPT, which can be transformed into SN38 through carboxylesterase-mediated de-esterification in vivo for therapeutic effect. However, CPT11 and SN38 only account for 5% of the human body. In addition, CPT11 does not perform well in clinical applications due to poor carboxylesterase activity in the human liver [128].

**Fig. 7:**
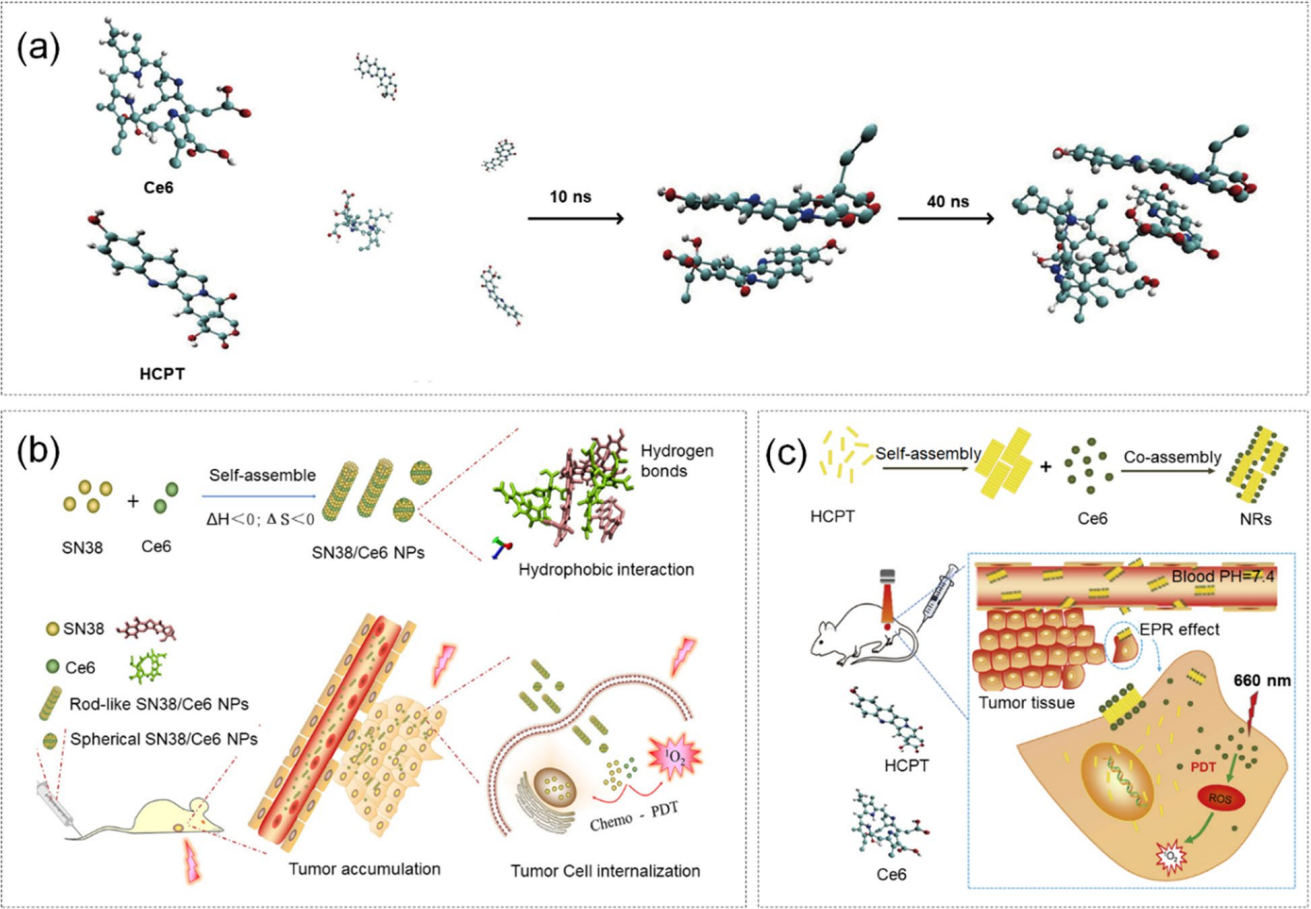
**a** MD simulation of the co-assembly of HCPT and Ce6 to prepare carrier-free HCPT/Ce6 nanorods. **b** Schematic diagram of self-assembled SN38-Ce6 NP assembly mechanism and chemotherapy-PDT combination therapy. Reproduced with permission from Ref [129]. **c** Schematic diagram of the application of chemo-photodynamic synergistic anti-tumor therapy. Reproduced with permission from Ref [76]

To address such problems, Zhao et al. [129]. self-assembled SN38 and Ce6, into a new type of nanoparticle SN38-Ce6. SN38-Ce6 NP was fabricated by a simple anti-solvent precipitation method. Researchers evaluated physicochemical property, combined chemotherapy-photodynamic antitumor effect, and ^1^O_2_ generation ability of SN38-Ce6. The driving force of the self-assembly process of SN38-Ce6 nanoparticles was revealed by chemical thermodynamics and visual images simulated by MD. The mechanism of assembly of SN38-CE6 NP and chemotherapy/PDT combination therapy is shown in Fig. 7 (b). The specific simulation was as follows, GROMACS 4.0.5 was used to perform MD simulation on Ce6 and SN38. The initial model was built. Energy minimization was applied using the steepest descent method. Then, researchers maintained the equilibrium state of temperature and pressure under the NPT ensemble. The study showed that SN38-Ce6 nanoparticles exhibited extremely high drug loading, high ROS generation efficiency, great cellular uptake rate and favorable tumor accumulation in different states. The laser can further enhance tumor cell killing ability with SN38-Ce6, confirming the good antitumor effect of chemical-photodynamic combined therapy.

The chemotherapeutic drug HCPT has a strong therapeutic effect on malignant tumors by inhibiting the ribozyme topoisomerase I pathway and rejoining the cleaved DNA strands. However, the lactone ring of HCPT is unstable under alkaline conditions and prone to hydrolysis [130, 131]. In addition, Ce6 is the second-generation PS which can efficiently generate ROS in Grand Lyon in the NIR irradiation section to penetrate deep tissues making it suitable in PDT. However, the weak target ability and bioavailability of Ce6 limit its therapeutic efficacy against cancer [132]. Zhao et al. [76]. self-assembled HCPT and Ce6 into a bifunctional nano-drug delivery system by reverse solvent precipitation method. Simultaneous introduction of MD simulation played an important role in analyzing the molecular interaction and self-assembly mechanism between Ce6 and HCPT. The assembly process and treatment plan of the HCPT/Ce6 Nanoparticles under MD simulation were shown in Fig. 7 (a, c). GROMACS 4.6.3 was used to carry out MD simulation of Ce6 and HCPT. The initial model was built using the CHARMM force field, and energy minimization was applied using the steepest descent method. Then, the equilibrium state of temperature and pressure was maintained under the NPT ensemble. HCPT/Ce6 NPs were highly stable in different situations, and a good cellular uptake and ROS production rate under laser irradiation of NPs were exhibited in the study. Compared with single chemotherapy or PDT, bifunctional HCPT/Ce6 NP had a significant synergistic tumor treatment effect.

#### Self-assembled IR780 nanomedicine

**Fig. 8:**
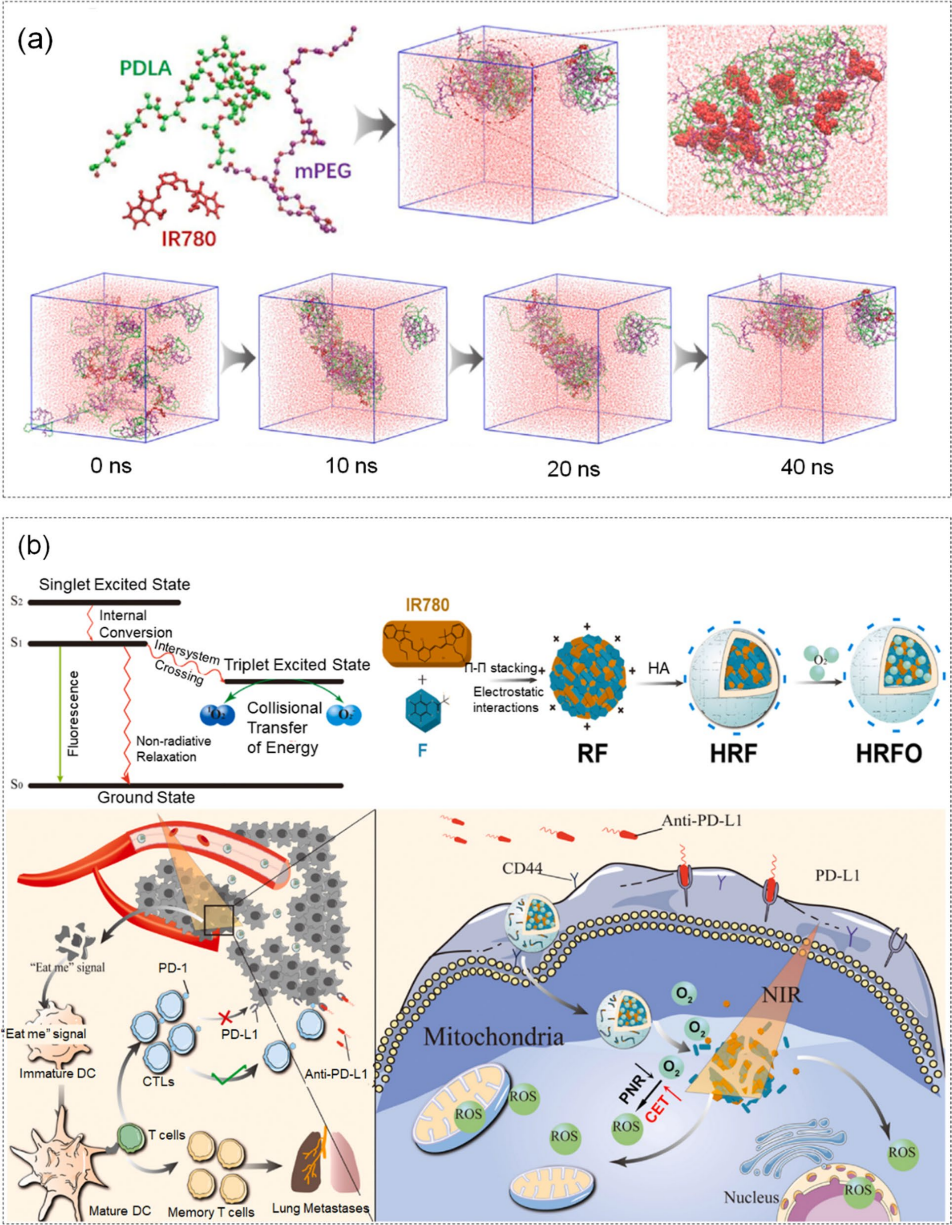
**a** Self-assembly process of IR780/mPEG-PDLA under MD simulation. **b** Schematic diagram of the assembly mechanism of IR780 nanoparticles and the treatment of tumor cells. Reproduced with permission from Ref [133]

Although the clinical application of organic photosensitizers in PDT is increasing, the current nano-encapsulation technology does not tend to convert from light energy to chemical energy.On this basis, Heng et al. [133]. designed a fluorocarbon-driven IR780 photosensitizer assembly through the interaction of IR780 iodide photosensitizer with fluorocarbon molecules, a photochemical switch used to unlock breast cancer suppression. And Hyaluronic Acid was decorated on the surface tprolong IR780 circulation and targeted tumors in vivo, and the intermolecular energy driving force was verified by systematic experiments and MD simulation to demonstrate the assembly process. IR780/MPEG-PDLA's self-assembly process and treatment case under MD simulation were shown in Fig. 8 (a, b).

During this stimulation, the point charge on each atom of the molecular model of pentafluorophenyl trifluoroacetate and IR780 was firstly obtained by Gaussian 09. The initial model was built using the GROMOS96 force field, and energy minimization was applied using the steepest descent method. Then, researchers maintained the equilibrium state of temperature and pressure under the NPT ensemble. It was found that the assembled ordered IR780 assembly could decode the energy conversion from light energy to chemical energy represented by ROS, suppressing the non-radiative relaxation represented by photothermal. In addition, fluorocarbons could also release oxygen to alleviate hypoxia and significantly enhance collisional energy transfer from ROS generation, supporting efficient PDT.

### MD simulation with chemotherapy

Chemotherapy is the most widely used anti-cancer treatment with reliable efficacy [134]. However, there are many limitations in the use of chemotherapy agents. Traditional chemotherapy drugs are non-targeted and could lead to systemic toxicity [135]. For example, doxorubicin (DOX), could inhibit the synthesis of Ribonucleic Acid (RNA) and DNA. It would be favorable to design drug carriers to deliver DOX to tumor sites specifically [136]. Paclitaxel (PTX) is microtubulin inhibitor with great tumor-suppressing effect, but its poor water solubility could cause serious side effects [137]. Another obstacle for single-drug regimen is the drug resistance [138]. MD simulation is a reasonable method to self-assemble two or more drugs into nanoparticles for better drug delivery and combined therapy [139, 140]. Besides, prodrugs are developed to increase drug stability, reduce adverse effects, and promote long-term efficacy.

#### Self-assembled PTX nanomedicine

**Fig. 9:**
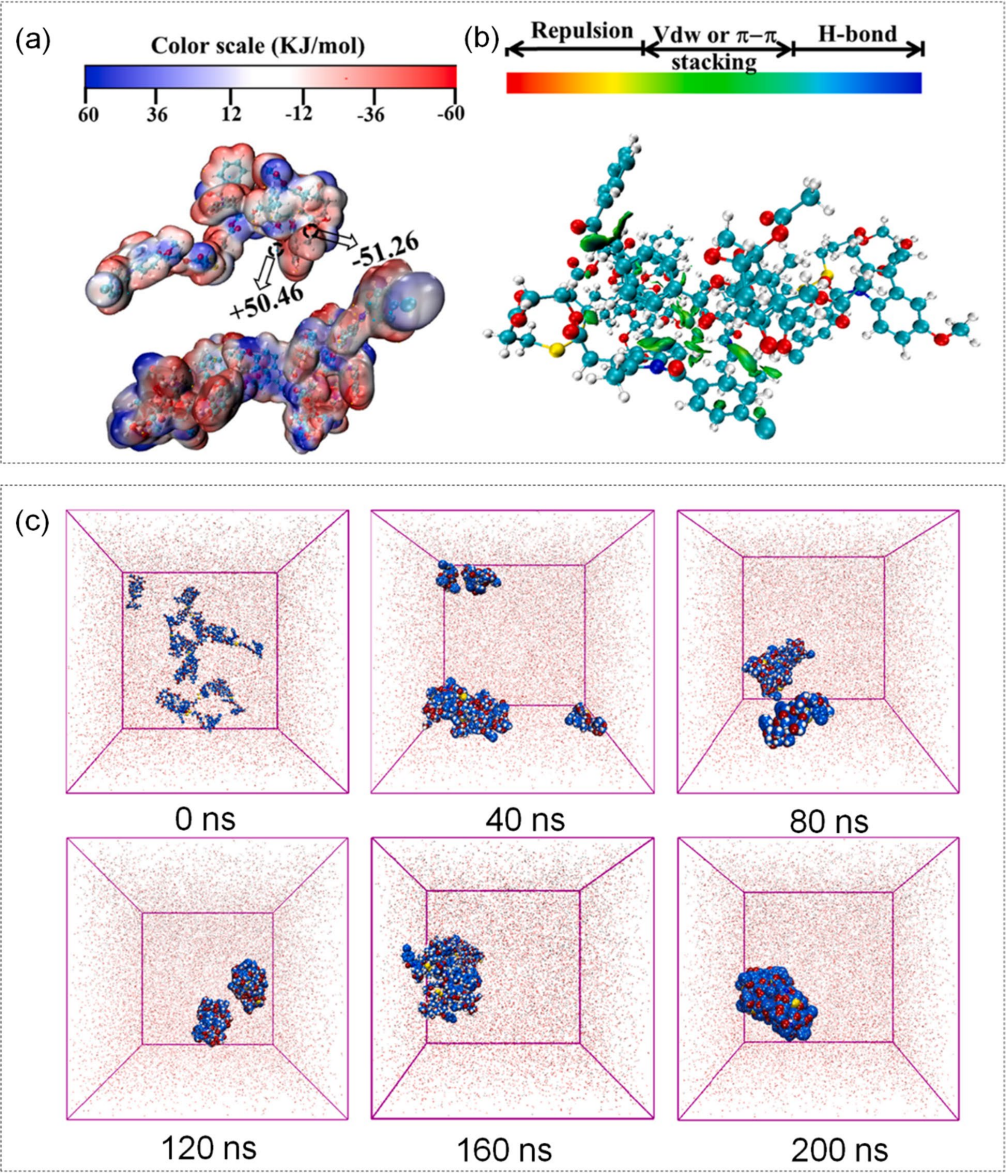
**a** CG structure of IND-S–S-PTX. **b** Interaction force analysis of IND-S–S-PTX. **c** MD simulation of the self-assembly process over time. Reproduced with permission from Ref. [143]

High expression of cancer resistance proteins is a main limiting factor for PTX application [141]. This phenomenon can be improved by combining indomethacin with PTX. Indomethacin (IND) is a potential MRP1 inhibitor [142]. Kang et al. [143]. connected IND with PTX via disulfide bond, and used MD simulation to construct conjugated IND-S–S-PTX as self-assembled nano-drug. The thick granular structure was shown in Fig. 9 (a, b). Disulfide bond is one of the most common chemical bonds in self-assembly nano-materials. The as prepared IND-S–S-PTX could target the high concentration of GSH in tumor microenvironment [144]. The specific simulation process was as follows: First, the initial model was built by the GAFF force field and the energy was minimised using the steepest descent method. Subsequently, the equilibrium state of temperature and pressure was maintained under the NPT ensemble, and the Coulomb and L-J potentials were used to describe the non-bonding interactions. And MD simulation was applied to imitate the self-assembly process over time. The simulation process of 0–200 NS was shown in Fig. 9 (c). It was found that IND-S–S-PTX NPs were well dispersed in water and sensitive to high levels of GSH in the tumor microenvironment with high stability. In addition, due to the existence of disulfide bonds, the NPs could also target tumor microenvironment through redox reactions with GSH. Thus INDS-S–S-PTX NPs could not only induce tumor cell apoptosis by PTX, but also reverse MDR by down-regulating the expression of MRP1 protein.

Berberine (BBR) is capable of accumulating in tumor cells with many prominent pharmacological effects, including antibacterial, antiviral, and antineoplastic activity [145–148]. BBR could combine with PTX to improve the anti-tumor therapeutic effect. Cheng et al. [149]. linked PTX and BBR via disulfide bonds to construct a GSH-sensitive conjugate (PTX-ss-BBR), and then applied MD simulation to explore the assembly mechanism of PTX-ss-BBR. PTX-ss-BBR conjugate force field parameters were generated by the GROMACS96 (53a6) force field that came with GROMACS 5.1.4. The initial model was built with GAFF force field and an equilibrium state of temperature and pressure. Energy minimization was carried out with the steepest descent method, followed by NVT and NPT to balance the system with position constraints. The study demonstrated that PTX-ss-BBR NPs could achieve a tumor microenvironment sensitive release. What’s more, the anticancer effect of PTX-ss-BBR NPs on A549 cells in vitro was enhanced compared with PTX due to the increase of ROS and the dissipation of mitochondrial membrane potential. In conclusion, the combined treatment of PTX and BBR improved the therapeutic ability of conventional chemotherapeutic drugs on tumor cells and MD simulation played an important role in this process.

**Fig. 10:**
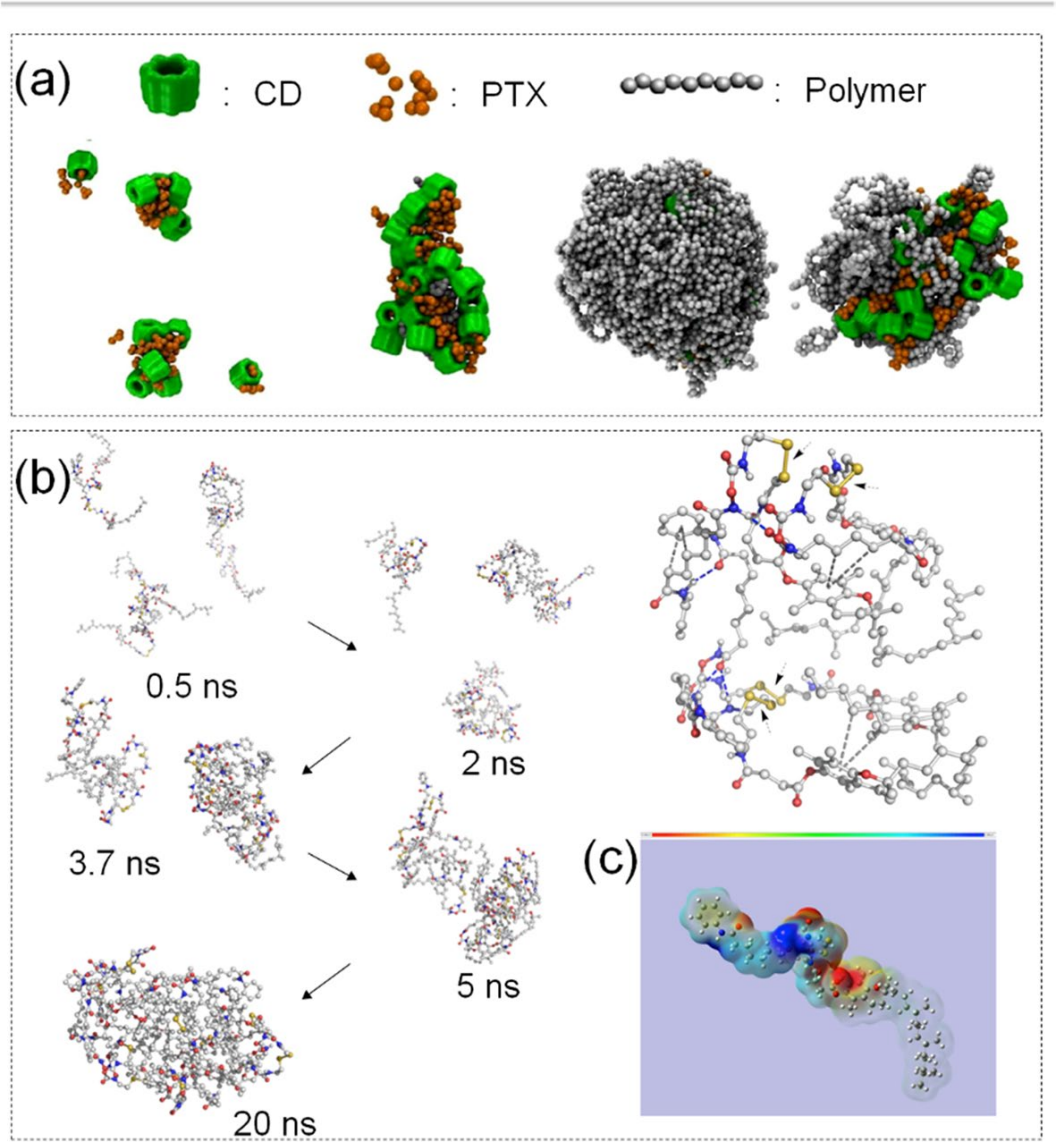
**a** Snapshots of MD simulations of three drugs with CD, PTX, Polymer. Reproduced with permission from Ref. [151] **b** Self-assembly of SAHA-SS-VE drug under MD simulation and its molecular interaction. SAHA-SS-VE molecule was indicated by gray ball-andstick model: red (O), blue (N), and yellow (s). **c** The electrostatic potential map of SAHA-SS-VE: red (electron enrichment region), blue (electron deficient region). Reproduced with permission from Ref. [153]

Cyclodextrin (CD) can form compounds with other hydrophobic drugs, the most common partner being the antineoplastic drug PTX to elevate water solubility of PTX [150]. Ran et al. [151]. investigated a novel nanodrug delivery system consisting of polymer-cyclodextrin (pCD) and polymer-paclitaxel (pPTX) for efficient PTX delivery to cancer sites. pCD and pPTX were expected to attach each other, and they would self-assemble into potent nanoparticles [152]. CG-MD simulation validated the successful assembly and stability of pPTX/pCD nanoparticles. The assembly process was shown in Fig. 10 (a). For the MD simulation part, researchers modelled pPTX/pCD for 50–100 ns and increased the bias potential, and then used the Martini force field for simulation. Each monosaccharide of the CD was represented by three beads, and non-bonding interaction parameters were selected from the Martini force field. For bonding interaction, the method of limiting the inter-bead distance was adopted. It was found that pPTX/pCD nanoparticles exhibited high stability during drug delivery. In addition, after reaching the tumor cells, drugs were spliced out of the nanoassembly and induced cancer cell death. In this study, MD simulation provide information about the morphology of each drug and how these components self-assemble.

To achieve better therapeutic effect, oncologists prefer to use drug cocktail treatments. However, the combination of current chemotherapeutic drugs can cause severe side effects. For example, the different water-solubility (hydrophobic and hydrophilic) among drugs leads to inconsistent biodistribution and reduced therapeutic efficacy [154]. Additionally, subcellular lysosomes, represented by hydrophobic-hydrophilic drug-loaded polymer nanoparticles, are blocked during nuclear and cell division, thereby reducing breast cancer spheroid formation. Based on this finding, San et al. [155]. described a unique cisplatin-initiated linear polymer self-assembly and explored the underlying mechanism of self-assembly by MD simulations. PTX and CDDP were chosen just because they have different mechanisms of action and high hydrophobicity and hydrophilicity respectively, resulting in different biological distribution. And this is how they ran the simulation process: Firstly, Guassian09 was used for quantum chemical QM calculation, the initial model was built and the equilibrium state of temperature and pressure was maintained under the NPT ensemble. The Interactions between non-bonded atoms were evaluated using van der Waals, energy minimization was carried out using the steepest descent method. The study found that the as prepared nanoparticles could enter tumor cell lysosomes and promote tumor cell death by inhibiting the formation of microtubules and delaying cell division. Furthermore, these polymer nanoparticles could significantly shrink 3D breast tumor spheroids within 24 h, promising an effective clinical treatment strategy for cancer patients. Consequently, this is another application case of MD simulation.

#### Self-assemerbled prodrugs nanomedicine

Prodrugs refer to structural modifications of existing drugs that are inactive or less active in vitro, but can release the active drug in vivo through enzymatic or non-enzymatic reactions [156]. With the development of technology, prodrug design has become attractive in drug development research. At present, prodrugs can not only alter tumor uptake efficiency, but also improve the water solubility and bioavailability of active drugs. Therefore, more and more scholars are paying attention to the application of prodrugs in tumor treatment [157].

Histone deacetylase (HDAC) is a genetic protein target that is highly expressed in tumor cell lines. HDAC inhibits histones, and induces cell division and death [158]. Vorinostat (SAHA) is a typical HDAC inhibitor that has been widely used to treat cutaneous lymphoma, but lacks efficacy in solid tumors [159]. Han et al. [153]. synthesized a redox-responsive prodrug SAHA-SS-VE with disulfide bonds by reaction, and then SAHA-SS-VE could be regarded as a novel SAHA nanomedicine. MD simulations were used to further test the assembly mechanism of SAHA-SS-VE into nanoparticles by nanoprecipitation. SAHA-SS-VE drug assembly process under 20 ns MD simulation and the electrostatic potential map of SAHA-SS-VE were shown in Fig. 10 (b, c). SAHA-SS-VE molecule was indicated by gray ball-andstick model: red (O), blue (N), and yellow (S). In the SAHA-SS-VE electrostatic potential diagram, red represents electron rich regions and blue represents electron deficient regions. The specific simulation was as follows. Calculated the electrostatic potential of SAHA-S–S-VE by Gaussian09 package. Built the initial model using the CHARMM force field, and energy minimization using the steepest descent method. Then, maintaining the equilibrium state of temperature and pressure under the NPT ensemble. It was found that free SAHA could be effectively released during redox reactions. SAHA-SS-VE/TPGS nanoparticles were functionalized with biocompatible D-a-tocopheryl polyethylene glycol succinate (TPGS) to accumulate in the tumor area and effectively inhibit tumor growth. In conclusion, SAHA delivery in solid tumors could be improved by this redox-reactive nanodrug delivery system which provided another idea for HDAC inhibitors in the treatment of solid tumors.

**Fig. 11:**
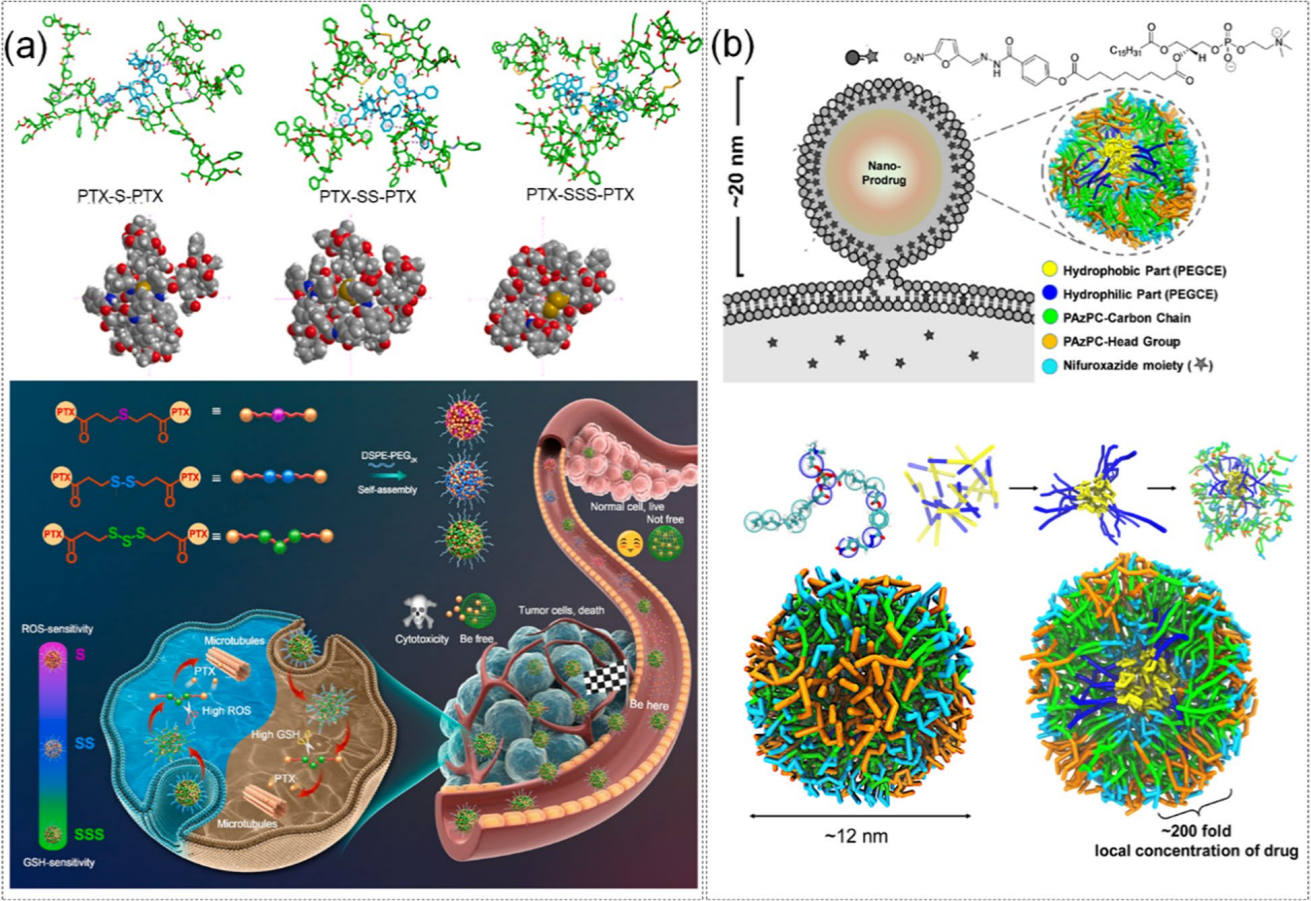
**a** Three molecular structures of PTX homodimers and their therapeutic options. Reproduced with permission from Ref. [163] **b** Schematic diagram of prodrug-NP interaction and CG-MD simulation of the molecular self-assembly process of Pro-nifuroxazide. Reproduced with permission from Ref. [162]

Pro-Nifuroxazide was first used as an antibiotic agent for diarrhea or colitis which reduces kinase phosphorylation in the bone marrow, resulting in the downregulation of the signal transducer and activator of transcription 3 (STAT-3) target gene mcl-1 [160, 161]. Santosh et al. [162]. designed a new nanodrug delivery system to improve the solubility of poorly soluble drugs and the inhibition of STAT-3, thereby enhancing the anti-cancer effect. MD simulation was used to explore the self-assembly process of nifuroxazide NP structures. Silica properties of pre-nifuroxazide, chemical properties of NPs, and intermolecular membrane interactions were explored through activity in cancer cells. Pro-Nifuroxazide NP interaction and molecular structure are shown in Fig. 11 (b). In this case, CG-MD simulation was used. Firstly, built a molecular structure model using the CHARMM force field, and umbrella Sampling with NAMD 2.9 to Compute Mean Force. Maintained the equilibrium state of temperature and pressure under the NVT ensemble. The results showed that the pro-nifuroxazide NPs required less time to achieve tumor cell suppression compared to the parental nifuroxazide. After assembly into NPs, the concentration of the drug in some parts of the tumor cells was significantly increased by about 240-fold. Nifuroxazide precursor NP had anticancer effects in vivo, with up to 400% growth inhibition. Through the treatment of pro-nifuroxazide NP, the level of transcription factor pSTAT-3 was significantly decreased which could inhibit the phosphorylation of STAT-3, effectively inducing cancer cells apoptosis.

In recent years, Sulfur bonds have been broadly applied in the research and development of self-assembled prodrugs due to their close to 90° bond angle and the characteristic that they could enhance the structural flexibility of prodrugs [164–167]. Besides, assembly with trisulfide bonds improves drug stability compared to disulfide and thioether bonds [168]. Yang et al. [163]. synthesized three PTX homodimer prodrugs with trisulfide, disulfide and thioether bond as the connecting bond. MD simulation of chemical structures and microscopic interactions of prodrug molecules was used for elucidating self-assembly mechanisms. In addition, an in-depth study of the differences among sulfide/disulfide/trisulfide bonds was utilized for elucidating the therapeutic effects of prodrug nanoparticles with different sulfur bonds on tumors. The structure and treatment plan of the three PTX and di-polygon were shown in Fig. 11 (a). The simulation process was as follows, the structures of PTX-S-PTX, PTX-SS-PTX and PTX-SSS-PTX were constructed based on GaussView 5 software. Then the charge parameters of Restrained ElectroStatic Potential (RESP) were fitted based on the Antechamber program, and the parameters of them were generated by the Gaff force field. The initial model was built by the Packmol program. The results showed that the prodrug nanoparticles assembled with trisulfide bonds exhibited faster drug release compared to the other two sulfur bonds. Because the trisulfide bond could promote the stability of the assembled colloid, thereby effectively promoting the self-assembly of the prodrug. At the same time, the trisulfide bond was also a new type of redox bond, which provided in-depth insights into the effect of sulfur bond on the nanoassembly of the original drug. In addition, the stronger electrostatic interaction of PTX-SSS-PTX itself would also improve the stability and safety of its self-assembled structure, and highlight the role of trisulfide bonds in redox dual-responsive nanoself-assembly.

**Fig. 12:**
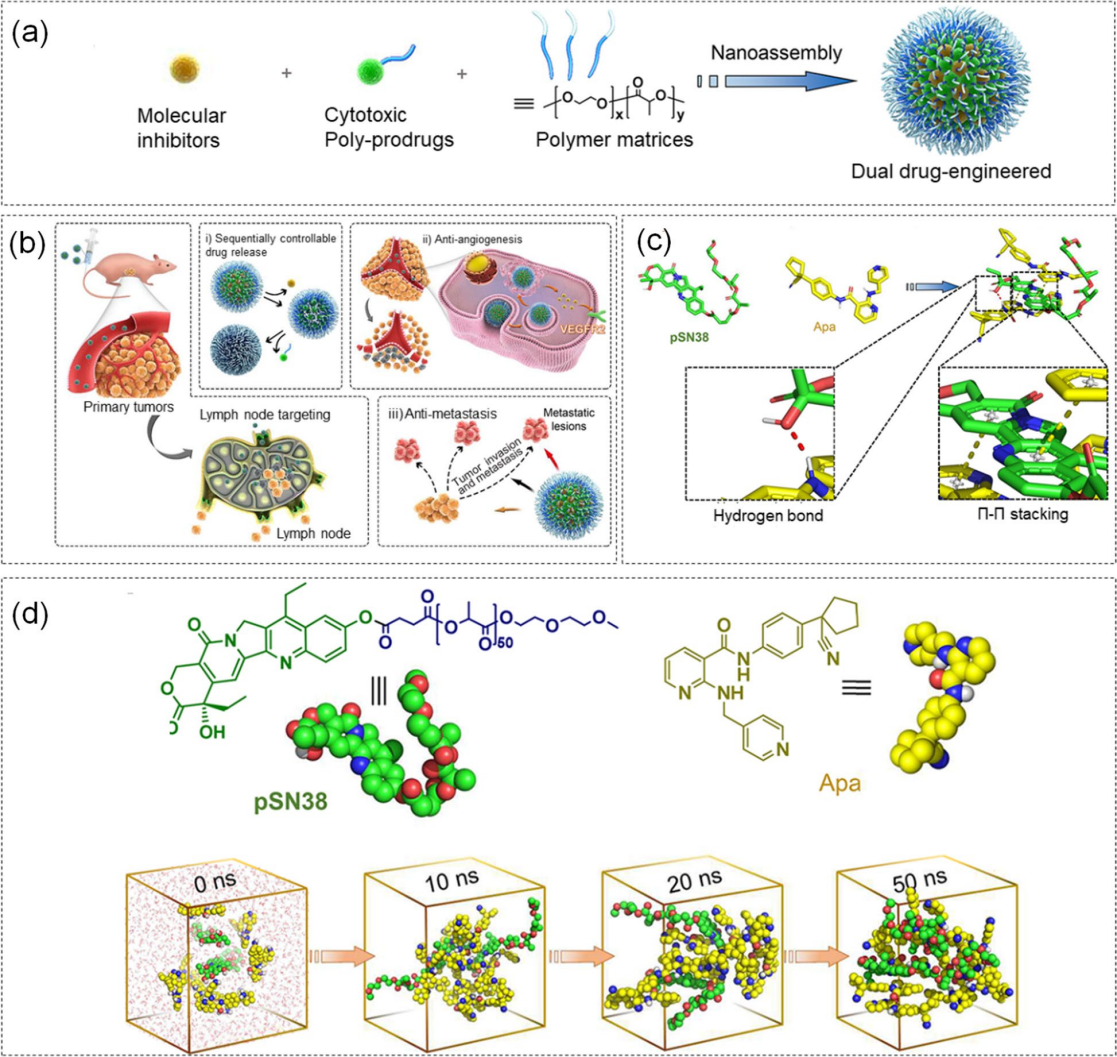
**a** Schematic illustration of the assembly of sTKI-pSN38 nanoparticles. **b** Tumor treatment regimen of sTKI-pSN38 nanoparticles. **c** Interaction between pSN38 and Apa during assembly. **d** The assembly process of pSN38 and Apa under MD simulation. Reproduced with permission from Ref. [174]

The rational pairing of molecular inhibitors TKIs with chemotherapeutic agents can achieve additive or synergistic effects to improve treatment outcomes [169–171]. Although continuous multiple drug delivery or segmented multiple drug delivery can enhance the antitumor activity of the drug, it will weaken the cytotoxicity of the drug molecule [172, 173]. To address this question, Han et al. [174]. developed a new protocol for incorporating cytotoxic nanoparticles, such as apatinib (Apa), hyperstabilized with a π-rich hydrophobic core with a polymeric SN38 precursor the drug (pSN38) binds to self-assemble a synergistic drug delivery system (sTKI-pSN38). 7-Ethyl-10-hydroxycamptothecin (SN38) was a potent DNA topoisomerase I inhibitor with an inhibitory effect on tumor cells [175, 176]. However, since SN38 was not easily to be encapsulated in polymeric nanoparticles, a pSN38 prodrug was constructed by hydrolyzing ester bonds using polylactic acid (PLA) as a promoter [177]. The synthesis and tumor treatment scheme of STKI-PSN38 nanometer particles were shown in Fig. 12 (a, b). The specific simulation was listed as follows. The structures of pSN38 and Apa were first optimized by setting B3LYP/6-31G* in the Gaussian09 software package, and the electrostatic potential was calculated. The initial model was built using the CHARMM force field, and energy minimization was performed using the steepest descent method. Then, they maintained the equilibrium state of temperature and pressure under the NPT ensemble. The interaction between PSN38 and APA and the assembly process of 50 ns under MD simulation were shown in Fig. 12 (c, d). Experiments found that sTKI-pSN38 treatment reduced intratumorally hypoxia, inhibited tumor cell growth, inhibited lymph node metastasis, and improved the therapeutic ability. Simultaneously, Non-covalently engineered multidrug nanotherapeutics were a novel tumor treatment option. It increased the targeting and effective therapeutic properties of the drug without complicating the drug delivery system.

#### Self-assembled DOX nanomedicine

Curcumin (Cur) is a chemical sensitizer which can reverse multidrug resistance (MDR) and inhibit the division of cancer cells [178]. The combination of Cur and DOX can reduce the cytotoxicity of DOX and achieve a more efficient therapeutic effect [179]. Based on this discovery, Samaneh et al. [180]. studied a new hexadic m-phenylene ethynylene (m-PE) macroing tubular assembly structure, to explore the ability of ten macrocyclic self-assembly nanotubes in chloroform and aqueous solvents. MD simulation was used to study the double transport of antineoplastic drugs Cur and DOX on hexamer nano-carrier self-assembly system. The specific simulation was as follows. Built the initial model using the CHARMM force field, and energy minimization using the steepest descent method. Then, maintain the equilibrium state of temperature and pressure under the NPT ensemble. The DOX loading on the self-assembled hexagonal surface was significantly increased in the presence of the Cur drug. This confirmed that the combination of Cur and DOX reduced cytotoxicity, and macrocyclic compounds could also be used to develop drug delivery systems which had better potential than CNTs nanostructures in drug delivery systems [181].

Researchers have confirmed that DOX combined with other drugs can reduce cytotoxicity, but which drug has the best adsorption effect on DOX has not been confirmed. Among the polymers, polylactic-glycolic acid (PLGA) has attracted much attention because of the hydrophobicity and hydrophilicity of lactic acid and glycolic acid. Both single-hydrophobic/hydrophilic anticancer drugs and hydrophobic-hydrophilic anticancer drugs can be captured into PLGA particles by nanoprecipitation [182]. At the same time, polyethylene glocalization (PEG) of PLGA can improve drug loading and prolong blood circulation, thereby reducing protein adsorption and producing "invisible" nanoparticles [183]. Riboflavin (RF) has anti-inflammatory properties and is important for the body's autoimmune function. loss of RF can lead to oxidative damage, cellular stress response and brain nerve dysfunction[184].

**Fig. 13:**
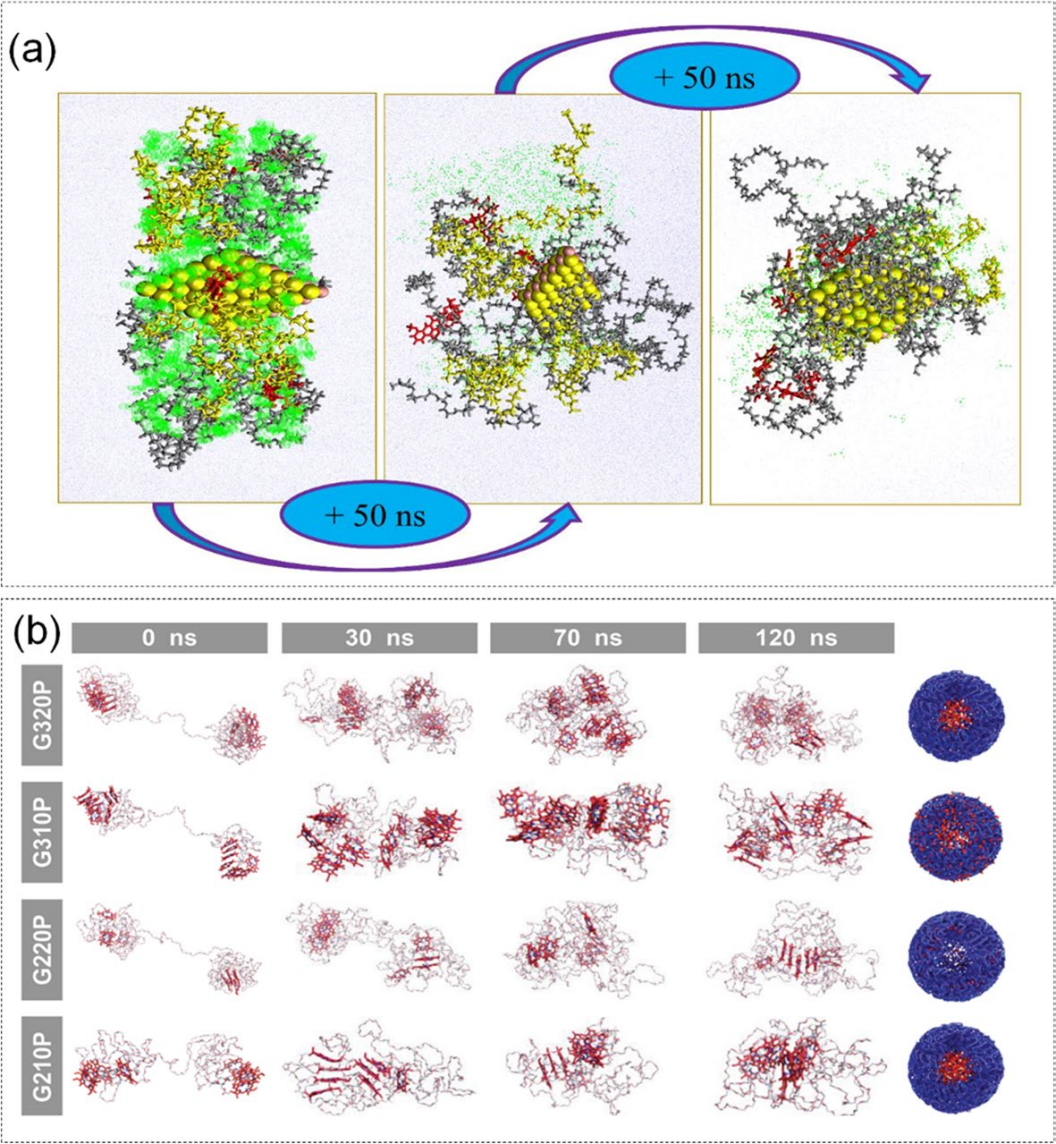
**a** MD simulation renderings of nanomedicines and micellar nanocarriers containing MoSe2. Reproduced with permission from Ref [185]. **b** Observation of the self-assembly process of G320P, G310P, G220P and G210P in MD simulation. Reproduced with permission from Ref. [186]

Based on this discovery, Maleki et al. [185]. studied the loading of DOX on nano-carriers composed of PLGA, polyethylene glycol and RF micelles (PLGA-PEG-RF) and metal second-stream compounds nanolayers by MD simulation. The self-assembly of PLGA-PEG-RF was studied by microfluidic method, and the nanoscale interaction was simulated by MD, which provided the mechanism of DOX packaging and PLGA-PEG-RF micellization. MD stimulation was used as a powerful tool. Firstly, the DFT method was used to optimize the original unit of the 2D structure through the software Gaussian 09. Then, the initial model was built using the OPLS-AA force field, and energy minimization was performed with the maintaining equilibrium state of temperature and pressure under the NPT ensemble. The experimental results showed that among the selected metal dichalcogenides, the combination of Molybdenum (IV) selenide (MoSe_2_) and DOX molecules was the best which made the lower contact area around the micelles to adsorb more DOX. Self-assembly process of adhesive nano was shown in Fig. 13 (a). Meanwhile, MoSe_2_ had a great influence on the stability and size of nanocarriers, which could improve the loading capacity of RF-targeted micelles. Therefore, this method was one of the best ways to improve the quality of micelles.

In biological applications, researchers propose to improve the stability of nanoparticles by encapsulating amphiphilic molecules in amphiphilic polymer micelles. Magnetic nanoparticles (MNPs), especially magnetite (Fe_3_O_4_) MNPs, have attracted much attention recently. Fe_3_O_4_ MNPs show unique characteristics and intelligent functions and are suitable for nanomedicine [187]. On the other hand, hydrophilic and hydrophobic drugs could hardly be delivered simultaneously, self-assembled drugs are needed to solve this problem. Danial et al. [188]. connected polyethylene glycol with a disulfide bond (PEG-SS-PCL) to simulate the self-assembly of PEG-PCL amphiphilic polymers, and then prepared biodegradable micelles with reductive response function for the delivery of DOX and superparamagnetic iron oxide nanoparticles (SPION). The stability and cytocompatibility of self-assembled nanoparticles were investigated in different environments by CG-MD. They first built the initial model using the Martini force field. Maintained the equilibrium state of temperature and pressure under the NPT ensemble. VMD software was used to visualize the results. It was found that the nanoparticles could be loaded into the core of SPION nano-micelles covered with oleic acid. Due to the existence of disulfide bonds, magnetic mPEG-SS-PCL/Fe_3_O_4_ nano-micelles showed redox response release. The micelles loaded with DOX had a low release rate in non-reduction environment, while in reduction environment, iron oxide nanoparticles could be released rapidly and frequently. In conclusion, these results revealed that reduction-sensitive nanogels loaded with DOX-SPION hold considerable biomedical prospects.

#### Self-assembled PEG–Ppa nanomedicine

Hydrophilic-lipophilic balance (HLB) plays a key role in the amphiphilic self-assembly of NPs, influencing their morphogenesis and biological effects [189, 190]. However, the tunability of HLB could determine the self-assembly behavior of NPs and has become a challenge in the drug delivery of NPsin HLB. Based on this finding, Zheng et al. [186]. used 2,2-bis (hydroxymethyl) propionic acid hyperbranched PEG-OH dendrimer as the hydrophilic unit and pyropheophorbide-a (Ppa) as the hydrophobic unit to synthesize amphiphilic molecules with adjustable HLB value. Four bis-MPA hyperbranched PEG–Ppa amphiphiles were synthesized with two PEG molecular weights and two generations of dendrimers. The stability of four amphiphilic self-assembled nanostructures was evaluated by MD simulation [191]. The four drugs under MD simulation were shown in Fig. 7 (b). The specific simulation procedure was like this: The initial structure was constructed in Molecular Operating. GAFF and energy minimization were applied using the steepest descent method. Then, maintained the equilibrium state of temperature and pressure under the NPT ensemble. The results showed that the nanoparticles with intermediate HLB value had better structural stability and tumor therapeutic ability [192].

### MD simulation with immunotherapy

Immunotherapy has become a powerful clinical strategy for the treatment of malignant tumors that controls the immune system to recognize and destroy tumor cells [193]. Tumor immunotherapy can effectively eradicate primary and metastatic tumors and inhibit tumor via the anti-tumor immune response produced by the host cells [194, 195]. Tumor elimination through T-cell-mediated immunotherapy (such as immune checkpoint blocking (ICB)) is a common strategy of immunotherapy. However, binding of programmed death receptor (PD-1) and its ligand (PD-L1) leads to T cell depletion, allowing tumor cells to evade host immune surveillance, resulting in low response rates for immunotherapy multiple tumors.

**Fig. 14:**
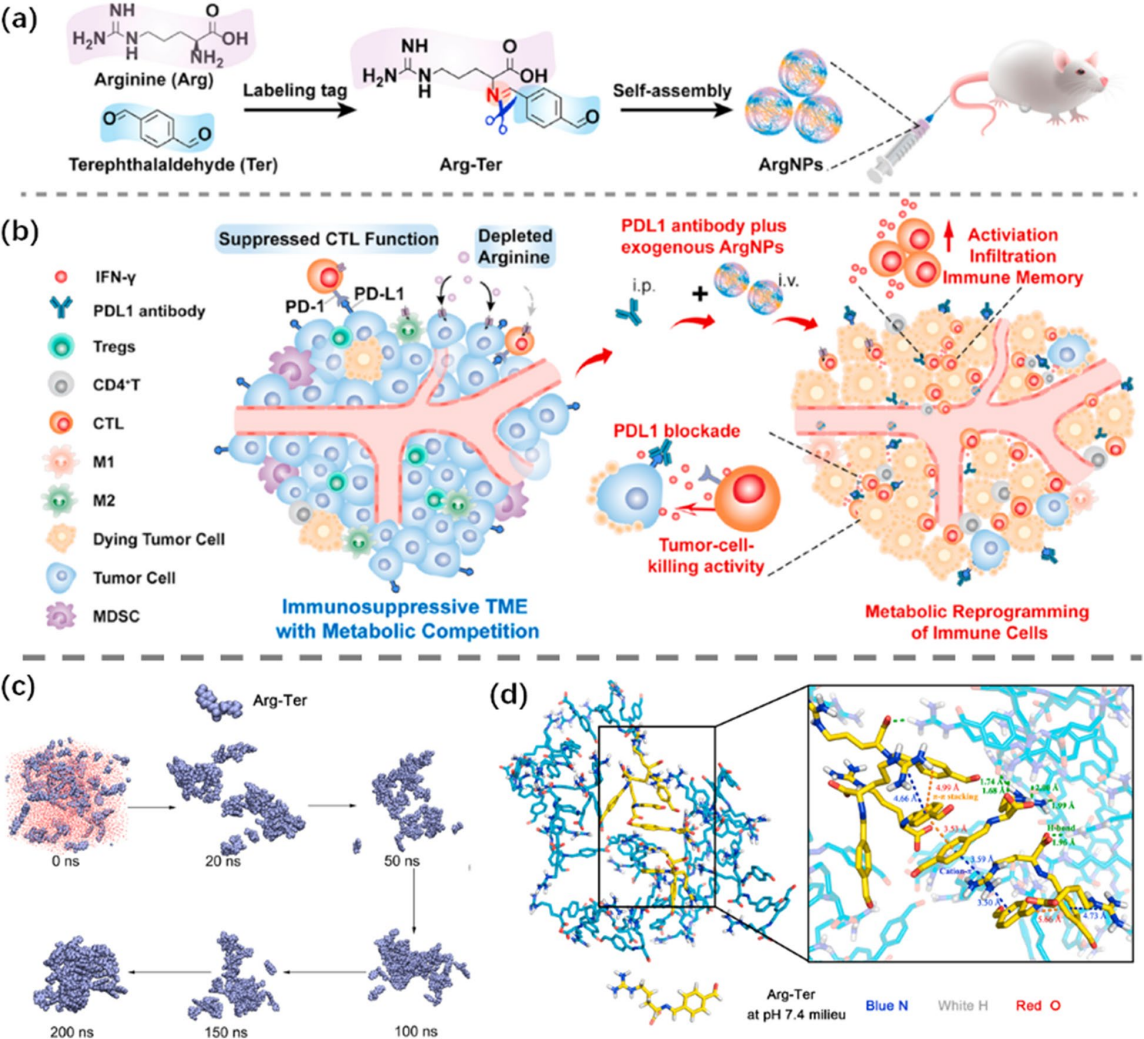
**a** Self-assembly process of ArgNP particles. **b** Schematic diagram of the mechanism of ArgNP metabolism enhancing immunotherapy. **c** Snapshots of the whole process of ArgNP formation in MD simulation. **d** Simulation of associative modes in ArgNPs. Reproduced with permission from Ref [200]

The efficacy of immunotherapy can be improved by combining immunotherapy with other strategies [196, 197]. Dynamic labeling of L-arginine metabolism that drives arginine assembly to metabolically enhance ICB therapy is critical for T lymphocyte activation and survival [198, 199]. However, due to low loading efficiency and rapid diffusion, hydrophilic L-arginine is difficult to deliver effectively into TME. In order to solve this phenomenon, Zhang et al. [200] selected aromatic aldehydes as dynamic tags to modify L-arginine, and designed a dynamic label-mediated self-assembly to generate nano arginine (ArgNP). The self-assembly process of ArgNP particles and the mechanism of metabolic enhancement of immunotherapy were shown in Fig. 14(a, b). In this simulation process, ArgNP assembly process was carried out under constant temperature and pressure by using the fastest descent method through Gromacs program; water molecules were analyzed by using tip3p model and Lincs algorithm for hydrogen bond analysis. In addition, the electrostatic interactions were calculated using the particle grid Ewald PME method. The V re-scale temperature coupling method was used to control the simulated temperature. The simulation of specific simulation process and combination mode was shown in Fig. 14(c, d). MD simulation further showed that hydrogen bond interaction, π-π stacking and cation-π interaction were important driving forces in ArgNP assembly process. ArgNP had good biocompatibility and could be further combined with aPDL 1 to enhance the inhibition of in situ tumor and lung metastasis. The combination of ArgNP could also promote the formation of memory T cells, which was of great significance for the treatment of tumor.

**Fig. 15:**
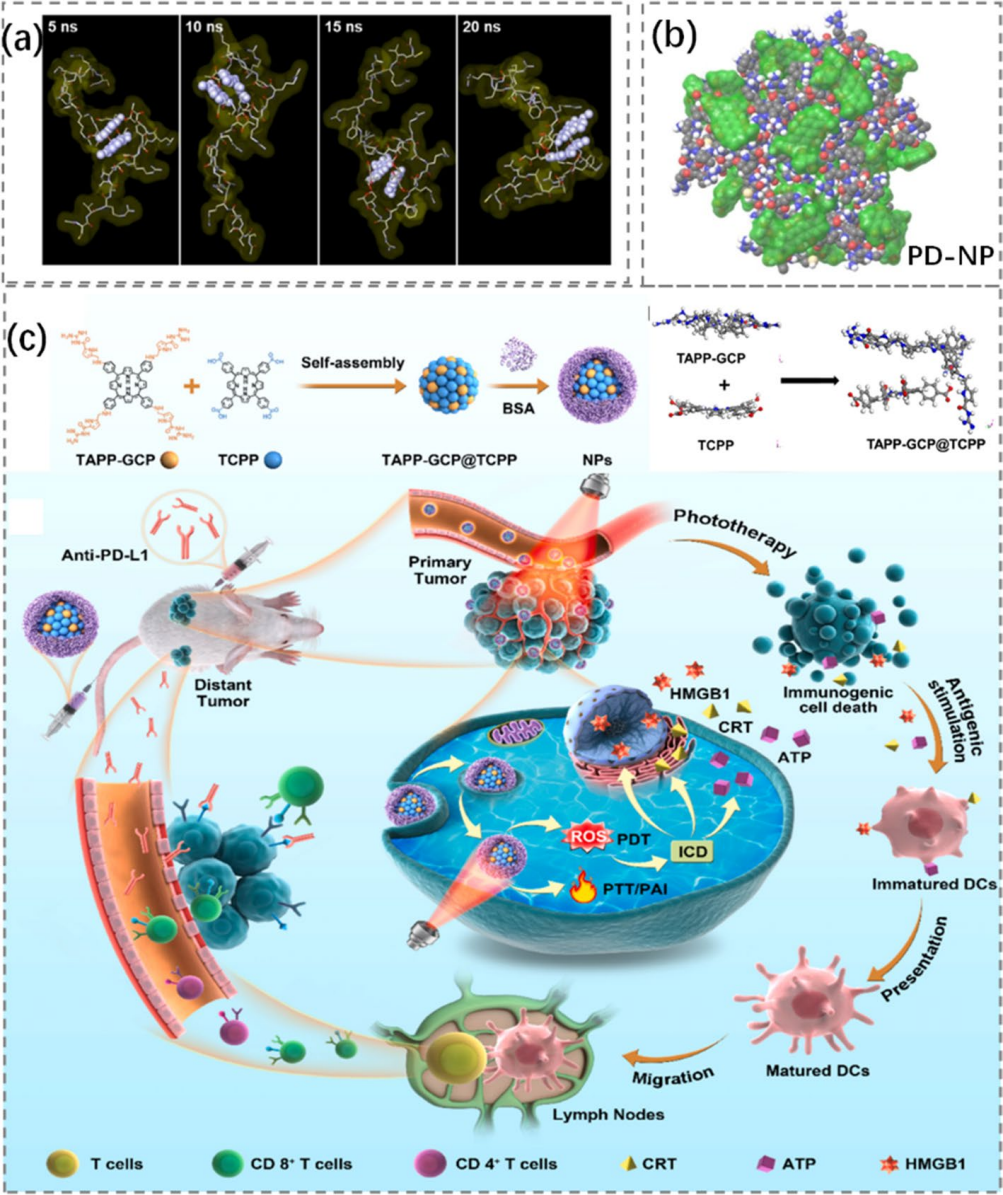
**a** MD simulation of PD-NP process II, and translucent green labeling of DOX molecular surface. **b** Structure diagram of whole atomic MD simulation results of PD-NP, in which the aromatic part of DOX is shown in light purple. Reproduced with permission from Ref [202]. **c** TAPP-GCP@TCPP Preparation of NPs, TAPP-GCP and TCPP molecules under MD simulation, and mechanism diagram of NPs phototherapy-induced immunotherapy. Reproduced with permission from Ref [201]

Immunogenic phototherapy has the potential to enhance tumor immunotherapy because of its favorable biocompatibility, no drug resistance and less invasive. However, its therapeutic effect is severely limited by the lack of optimal photosensitizers and effective delivery of photosensitizers. For example, the strong hydrophobicity of photosensitizer porphyrins makes it difficult to produce well-dispersed nanoparticles. To solve such problems, Kui et al. [201] constructed TAPP-GCP@TCPP@BSA nanoparticles by self-assembly of porphyrin derivatives (TAPP-GCP), meso-tetris (4-carboxy-phenyl-porphyrin) (TCPP) and bovine serum albumin (BSA). TAPP-GCP@TCPP The preparation of NPs and the mechanism of NPs phototherapy-induced immunotherapy were shown in Fig. 15(c). The main driving forces of self-assembly were found to be electrostatic, hydrogen bonding and π-π interaction by MD simulation. Laser irradiation of nanoparticles could induce tumor cytotoxicity and immunogenic cell death (ICD), thereby activating the specific antitumor killing effect of T cells in breast tumor models. This study provided a potential combination therapy for tumor photoimmunotherapy.

Cancer immunotherapy in combination with ICBs and chemotherapy drugs has also made significant progress. However, this combination regimen has the severe toxicity and low response rate of both drugs. In order to overcome this problem, Moon et al. [202] proposed coupling functional peptides composed of anti-PD-L1 peptide and pepsin B specific lytic peptide with DOX, and preparing prodrug nanoparticles (PD-NPs) through intermolecular interaction. The MD simulation of constant temperature and constant pressure under NPT ensemble was carried out. The Desmond module was used to study the molecular stacking mode and the force field parameter of OPLS3e was used. Elimination of excess conformational isomers by maximum atomic deviation cutoff. The structural molecules and simulation processes of PD-NP were described in Fig. 15(a, b). It was found that PD-NP was highly accumulated in tumors, which not only converted immunosuppressive TMB into immunoresponsive tumor TMB through DOX-mediated ICDs, but also enhanced the existing anti-tumor immune response of T lymphocytes by blocking PD-1/PD-L1 interaction mediated by anti-PD-L1 peptide.

## Conclusions and future perspectives

The rapid development of nanotechnology leads to the birth of with good application prospects in both research and clinical utilization, nanomedicine, especially the self-assembled nanodrugs have been a hot area of research recently. Traditional experimental methods are difficult to prove the achievement of self-assembly and could hardly give microscopic explanations of the mechanisms behind. As computer science is rapidly advancing, MD simulation technology has become a scientifically valid way to study self-assembly as a supplement to theoretical methods and experimental explorations. Consequently, an increasing number of scholars are applying MD simulation to the research of self-assembly nanomedicine.

In this review, we listed the application of MD simulation in self-assembled nanomedicine and it turns out that most of these studies belong to drug development area. MD simulations provide a unique perspective for exploring the mechanism of the self-assembly process, using a variety of methods and tools to analyze and simulate nanomedicines at different scales. Broadly speaking, MD simulation could be widely used for nanomaterial with sizes from angstroms to centimeters, and the calculations could be accurate to seconds, even femtoseconds. The MD simulations discussed in this review mainly focus on the atomic scale, basic methods, force fields, optimization and expectations. In the self-assemble field, MD simulations could not only provide a generalization of vast amount of accurate data and information, but also show the trend of self-assembly. When MD simulations are applied to self-assemble simulation, the pressure, temperature and other properties would be considered in the analysis. A summary of previous studies suggested that hydrophobic interactions (π-π stacking) and hydrogen bonding are the main drivers of the molecular self-assembly process, which broaden research thoughts for exploring the self-assemble mechanism of more complex nano-drug delivery systems.

Taken together, this review demonstrated self-assembly nanotheranostics based on MD simulations. We think reasonable self-assembly is a drug that interacts with other drugs or molecules in a way that matches them structurally; In terms of function, different self-assembled drugs or components can not only inherit the physical and chemical properties of the molecules themselves, but even achieve the synergistic effect between different drug molecules or components. They can also facilitate further modification to improve the targeting or biological safety and other functions. With the increasing requirement, the micro-level interaction between drug molecules should be known. In this case, MD simulations are highly recommended. However, there are still limitations for MD simulations to be widely used.

Firstly, drug molecules are complex elements in three dimensions. The first step of MD simulation is to obtain the stereoscopic structure of molecules to be analyzed. This process is very complicated and should be accomplished in advance. For biomolecules with definite structures such as proteins and nucleic acids, it is usual practice to directly use the structure measured experimentally as the starting structure of the model, such as the molecular structure measured by X-ray crystal diffraction or nuclear magnetic resonance spectroscopy. For amorphous polymers, the structure of repeating units can be constructed first, and then the repeating units can be connected according to certain rules to build the polymer chain structure. The configurations of small organic molecules in the system can also be calculated by quantum chemistry. If the molecule is complex, you can run a single molecule simulation for a few picoseconds and then take the configuration as the equilibrium molecular configuration.

Secondly, in MD simulation process, complex environmental conditions often increase the difficulty of simulation. Some conditions reached during simulation are difficult to achieve in practical application, such as high temperature, high pressure, etc. And because it takes a lot of computing power, the MD simulation is generally performed at nanosecond level. When it comes to more precise analysis, the cost of money and time would become a burden. With the rapid improvement of computing speed and the rapid development of parallel technology, many computer hardware facilities with superior performance and mature molecular dynamics software have been developed, which alleviates the time cost of MD to a certain extent, making MD simulation an increasing technology in the simulation process of nano drugs. At the same time, for the study of some biological systems, the whole atomic MD simulation faces great challenges due to the huge workload. An effective method is to use the coarse-grained model to realize the theoretical study of biological macrosystems. The coarse-grained model has greatly expanded the scale of computer simulation by ignoring the atomic details and treating several atoms or molecules as a coarse-grained particle to establish a coarse-grained model of biological macrosystems.

Lastly, some parameters including temperature, pressure and energy should be converted into real attributes. While may lead to errors in the actual situation, and proposing a new MD simulation algorithm is a great challenge. Therefore, it is necessary to combine MD simulation and experiment to modify the conditions of MD simulation based on the experimental results. In fact, the characterization and testing of the experiment require a certain amount of time, during which amounts of changes have taken place in the microscopic configuration. Therefore, both the macro variables measured in practice and the MD simulation can be regarded as an average of the microscopic states, so that the MD simulation is also of great reference value for the experimental verification of nanosecond scale.

Self-assembly technology will become one of the most extensive drug synthesis techniques in the near future. But before that, a clearer understanding of drug properties is required. Thus, in the follow-up work, researchers could connect drug properties with therapeutic mechanisms, using MD simulation and experimental exploration to verify the therapeutic value of self-assembly. Meanwhile, another future direction of MD simulation is multiscale simulation, especially those based on CG-MD systems, which extend atomic simulations to a longer time scale. However, during coarse-grained simulation, the mean degree of freedom is difficult to determine. Besides, head computing is also a hotpot because it offers possibilities to quickly calculate whether two drugs can be combined, thereby greatly shortening the experimental time, and promoting the development of self-assembly. It is believed that with the advancement of computer technology and simulation technology, MD will be widely applied in more fields.

## Data Availability

Not applicable.

## Abbreviations

MD: Molecular dynamics
DNA: Deoxyribonucleic acid
PTT: Photothermal therapy
NIR: Near-infrared
PDT: Photodynamic therapy
CDT: Chemodynamic Therapy
CG: Coarse-grained
AMBER: Assisted model build-ingand energy refinement
OPLS: Optimized potentials for liquid simulations
GROMOS: Groningen molecular simulation
CHARMM: Chemistry at harvard macromolecular mechanics
NVT: Canonical ensembles
NPT: Isothermal and isobaric ensembles
GROMACS: Groningen machine for chemical simulations
NAMD: Nanoscale molecular dynamics
LAMMPS: Large-scale atomic/molecular massively parallel simulator
CVFF: Consistent valence force field
GAFF: Generation Amber Force Field
ILAA NHCs: Iodine (I_2_)-supported acetylated amylose nanohelix clusters
AA: Acetylated amylose
ICG: Indocyaninegreen
AIE: Aggregation-induced emission
TFM: Compound
PA: Photoacoustic
ROS: Reactive oxygen species
GNR: Gold micro-nanorods
Ce6: Chlorin e6
H_2_O_2_: Hydrogen peroxide
GSH: Glutathione
GPX4: GSH peroxide 4
•OH: Highly oxidized hydroxyl radicals
TEM: Tumor microenvironment
CPT: Camptothecin
CPT11: Irinotecan hydrochloride
HCPT: Hydroxycamptothecin
DOX: Doxorubicin
RNA: Ribonucleic acid
PTX: Paclitaxel
IND: Indomethacin
BBR: Berberine
CD: Cyclodextrin
Pcd: Polymer-cyclodextrin
pPTX: Polymer-paclitaxel
HDAC: Histone deacetylase
SAHA: Vorinostat
TPGS: D-a-tocopheryl polyethylene glycol succinate
STAT-3: Signal transducer and activator of transcription 3
RESP: Restrained ElectroStatic Potential
Apa: Apatinib
SN38: 7-Ethyl-10-hydroxycamptothecin
PLA: Polylactic acid
Cur: Curcumin
MDR: Multidrug resistance
m-PE: M-phenylene ethynylene
PLGA: Polylactic-glycolic acid
PEG: Polyethylene glocalization
RF: Riboflavin
MoSe2: Molybdenum (IV) selenide
MNPS: Magnetic nanoparticles
SPION: Superparamagnetic iron oxide nanoparticles
Ppa: Pyropheophorbide-a
HLB: Hydrophilic-lipophilic balance
ICB: Immune checkpoint blocking
PD-1: Programmed death receptor
BSA: Bovine serum albumin
ICD: Immunogenic cell death
Gem: Gemcitabine
FA: Folic acid
PGA: Polyglutamic acid
